# PBLD Orchestrates the STING‐Mediated Antiviral Immune Response and Autoimmune Diseases

**DOI:** 10.1002/advs.202514512

**Published:** 2025-11-08

**Authors:** Peili Hou, Hongchao Zhu, Xiaonan Sun, Ni Zhang, Song Wang, Xuexing Zheng, Xiaoyun Wang, Yueyue Feng, Fuzhen Zhang, Xingyu Li, Rui Li, Xiaomeng Wang, Yuanyuan Han, Jun Wang, Chuanhong Wang, Xiaoyang Yao, Hongmei Wang, Hongbin He

**Affiliations:** ^1^ Ruminant Diseases Research Center, Key Laboratory of Animal Resistant Biology of Shandong College of Life Sciences Shandong Normal University Jinan Shandong 250358 China; ^2^ Department of Rheumatology the Second Hospital of Shandong University Jinan Shandong 250033 China; ^3^ Department of Clinical Laboratory Shandong Provincial Hospital Affiliated to Shandong First Medical University Jinan Shandong 250021 China; ^4^ Department of Clinical Laboratory the Second Hospital Cheeloo College of Medicine Shandong University Jinan Shandong 250033 China

**Keywords:** autoimmune disease, autophagic degradation, innate immunity, PBLD, STING

## Abstract

Precise regulation of stimulator of interferon genes (STING) expression is critical for maintaining immune homeostasis and preventing autoimmune disorders. In this study, phenazine biosynthesis‐like domain‐containing protein (PBLD) is identified as a key modulator of the STING‐dependent antiviral type I interferon (IFN) response by suppressing coiled‐coil domain‐containing protein 50 (CCDC50)‐mediated selective autophagic degradation of STING. Notably, viral infection downregulates PBLD expression through two distinct mechanisms: transcriptional suppression via reduced transcription factor EB (TFEB) activity, and post‐translational degradation through an enhanced membrane‐associated RING finger protein 2 (MARCH2)‐mediated ubiquitin‐proteasome pathway. Together, these mechanisms establish a negative feedback loop that facilitates viral immune evasion. Moreover, *Pbld*‐deficient mice exhibit increased susceptibility to human adenovirus type 4 (HAdV‐4) infection compared with their wild‐type (WT) littermates. Importantly, *Pbld‐*deficiency in the 2,6,10,14‐tetramethylpentadecane (TMPD)‐induced lupus mice model attenuates STING expression and diminishes autoimmune phenotypes. Clinically, PBLD expression is elevated in patients with systemic lupus erythematosus and positively correlates with STING‐driven type I IFN signaling. Taken together, PBLD plays a dual role in STING‐mediated innate immunity against viral infection and autoimmunity, highlighting its potential as a therapeutic target for both antiviral infections and autoimmune diseases.

## Graphical abstract

During viral infection, PBLD expression is downregulated, thereby subverting host antiviral defenses and promoting viral replication. This study identifies PBLD as a positive regulator of STING‐induced type I interferon activation and delineated the molecular network underlying PBLD‐mediated antiviral immunity and autoimmune pathogenesis, highlighting its potential as a therapeutic target for both antiviral infections and autoimmune diseases.

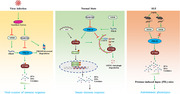

## 1 Introduction

Innate immunity provides the first line of defense against pathogenic microorganisms. Pattern‐recognition receptors, including Toll‐like receptors, C‐type lectin receptors, RIG‐I‐like receptors (RLRs), NOD‐like receptors, and cyclic GMP‐AMP synthase (cGAS), detect microbial components and regulate immune responses to ensure rapid pathogen clearance while maintaining immune homeostasis to avoid adverse effects.^[^ ^1^, ^2^ ^]^ Among these, the cGAS–stimulator of interferon genes (STING) signaling pathway is a vital component of the innate immune system, sensing cytosolic double‐stranded DNA (dsDNA) derived from both foreign and self‐DNA.^[^ ^3^ ^]^ Upon binding to DNA, cGAS synthesizes the second messenger cyclic GMP‐AMP, which activates STING. Subsequently, activated STING dimerizes and translocates from the endoplasmic reticulum (ER) to the ER‐Golgi intermediate compartment and Golgi apparatus, where it recruits and activates TANK‐binding kinase 1, thereby activating the transcription factor IRF3 and NF‐κB to induce the production of type I interferons (IFN) and other cytokines. Finally, secreted type I IFNs initiate the JAK‐STAT signaling cascade, upregulating IFN‐stimulated genes (ISGs) to establish an antiviral state.^[^ ^4^, ^5^ ^]^

The cGAS–STING signaling pathway, as a critical mechanism for inducing innate immune defenses, must be tightly regulated to maintain proper immune function. STING (also known as TMEM173), a key adaptor protein in the cGAS‐dependent DNA‐sensing pathway, plays a central role in driving antiviral immunity. Nevertheless, viruses have evolved numerous strategies to evade this defense system. For instance, several viral proteins—including human papillomaviruses type 18 oncoproteins E7, severe acute respiratory syndrome coronavirus 2 open reading frame 3a, human immunodeficiency virus 2 viral protein X, and Kaposi's sarcoma‐associated herpesvirus viral IFN regulatory factor 1—have been shown to selectively inhibit STING‐mediated innate immunity.^[^ ^6^ ^]^ Moreover, growing evidence indicates that dysregulation of the STING‐mediated signaling pathway is implicated in the pathogenesis of autoimmune diseases such as systemic lupus erythematosus (SLE). The Myb‐like, SWIRM, and MPN domains 1 and immunity‐related GTPase M protein have been identified as critical modulators of this pathway.^[^ ^7^, ^8^, ^9^, ^10^ ^]^ A deeper understanding of the master regulatory switches and molecular mechanisms controlling STING signaling will aid in developing therapeutics for viral infections and autoimmune diseases.

Phenazine biosynthesis‐like domain‐containing protein (PBLD) is an isomerase expressed in multiple tissues.^[^ ^11^ ^]^ Accumulating evidence indicates it plays a tumor‐suppressive role in various cancers.^[^ ^11^, ^12^ ^]^ Further studies have demonstrated the association of PBLD with tumorigenesis‐related signaling pathways, including angiogenesis through the VEGF–VEGFR2 signaling pathway, and attenuation of intestinal inflammation via the suppression of NF‐κB‐associated signaling pathways.^[^ ^13^, ^14^ ^]^ Our recent studies have revealed a critical role of PBLD in antiviral immunity. Specifically, during RNA virus infection, PBLD enhances antiviral innate immunity through the p53–USP4–MAVS axis.^[^ ^15^ ^]^ PBLD also stabilizes phospho‐IKKβ by antagonizing motif‐containing 21‐mediated degradation, thereby potentiating NF‐κB activation and IFN‐I production to protect against viral infection in both cellular and mouse models.^[^ ^16^ ^]^ Furthermore, PBLD promotes transcriptional phosphorylation of IRF3 at S385/386 to boost virus‐induced type I IFN production.^[^ ^17^ ^]^ However, whether PBLD participates in cGAS–STING‐mediated antiviral immunity or in autoimmune disease pathogenesis remains unknown.

In this study, we found that PBLD functions as a critical positive regulator of STING‐mediated type I IFN signaling by suppressing its the selective autophagic degradation of STING. Notably, we demonstrate that viral infection suppresses PBLD transcription via downregulation of transcription factor EB (TFEB) and promotes PBLD protein degradation through upregulation of the membrane‐associated RING finger protein 2 (MARCH2)‐mediated ubiquitin‐proteasomal pathway, thereby facilitating viral immune evasion and enhancing viral replication. Moreover, *Pbld* deficiency in 2,6,10,14‐tetramethylpentadecane (TMPD)‐induced lupus mouse models leads to diminished STING expression levels and attenuated autoimmune phenotypes. Clinically, PBLD expression is elevated in patients with systemic lupus erythematosus (SLE) and correlates positively with the type I IFN signature. Collectively, our findings indicate that PBLD represents a promising therapeutic target for both antiviral infections and autoimmune diseases.

## 2 Results

### 2.1 PBLD‐Deficiency Attenuates the cGAS‐STING‐Mediated Antiviral Immune Response

**Figure 1:**
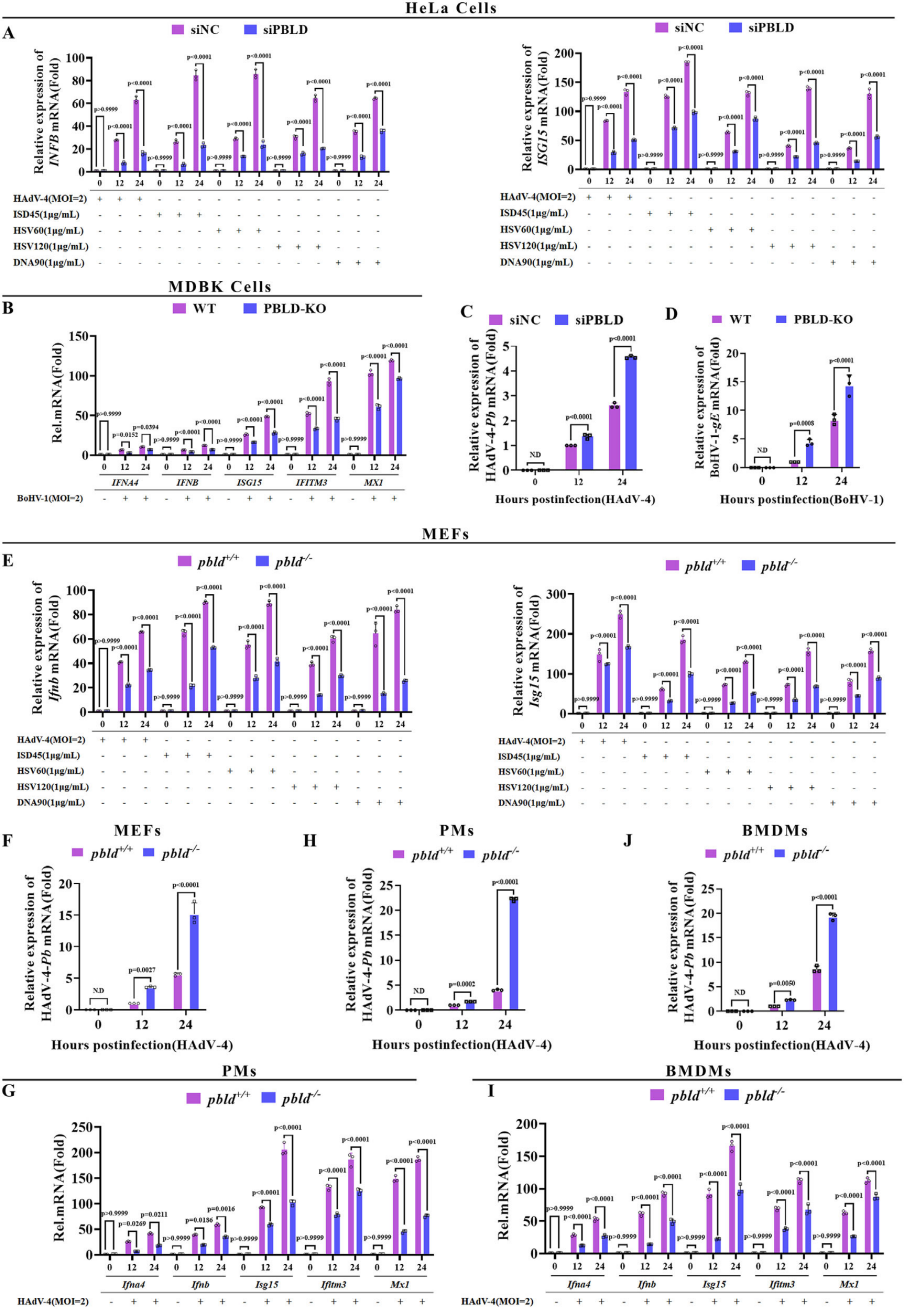
PBLD deficiency attenuates DNA virus‐ or interferon‐stimulated DNA (ISD)‐induced antiviral type I IFN response. A) RT‐qPCR analysis of the mRNA levels of *IFNB* and *ISG15* in control (siNC) and PBLD‐silenced cells (siPBLD) following HAdV‐4 infection or ISD45 (1 µg mL^−1^), HSV60 (1 µg mL^−1^), HSV120 (1 µg mL^−1^), and DNA90 (1 µg mL^−1^) transfection for the indicated time points. B) RT‐qPCR analysis of IFNs and ISGs mRNA levels in wild‐type (WT) and PBLD‐knockout (PBLD‐KO) MDBK cell lines infected with BoHV‐1 for the indicated time points. C,D) RT‐qPCR analysis of the mRNA expression of the HAdV‐4 *Pb* gene in siNC and siPBLD HeLa cells infected with HAdV‐4 (MOI = 2) **(C)**, and of the BoHV‐1 *gE* gene in WT and PBLD‐KO MDBK cells infected with BoHV‐1 (MOI = 2) **(D)** for indicated time points, respectively. E) RT‐qPCR analysis of *Ifnb* and *Isg15* mRNA levels in *Pbld^+/+^* or *Pbld^−/−^* MEFs infected with HAdV‐4 or stimulated with ISD45 (1 µg mL^−1^), HSV60 (1 µg mL^−1^), HSV120 (1 µg mL^−1^), and DNA90 (1 µg mL^−1^). F) RT‐qPCR analysis of the mRNA expression of HAdV‐4 *Pb* gene in *Pbld^+/+^* or *Pbld^−/−^* MEFs infected with HAdV‐4 for the indicated time points. G–J) RT‐qPCR analysis of IFNs, ISGs, and HAdV‐4 *Pb* gene mRNA levels in PMs (G, H) and BMDMs (I, J) from *Pbld^+/+^* or *Pbld^−/−^* mice after HAdV‐4 infection for the indicated time. Data in (A‐J) are presented as mean ± S.D., two‐way ANOVA; n = 3 biological independent experiments.

To determine whether PBLD regulates the cGAS–STING‐mediated signaling, we examined its role in DNA virus‐ or DNA‐mimic‐triggered type I IFN responses using siRNA‐mediated knockdown in HeLa cells and sgRNA‐mediated knockout (KO) in MDBK cells. As shown in **Figures** **1**A and S1A (Supporting Information), PBLD knockdown in HeLa cells markedly downregulated type I IFN (*IFNA, IFNB*) and ISGs (*ISG15, IFITM3*, and *MX1*) expression following stimulation with human adenovirus type 4 (HAdV‐4) or IFN‐stimulated DNA (ISD45, HSV60, DNA90, and HSV120). Consistently, the mRNA expression of type I IFNs and ISGs was decreased in PBLD‐KO MDBK cells in response to bovine herpesvirus 1 (BoHV‐1) infection (Figure 1B). Furthermore, under HAdV‐4 or BoHV‐1 infection, knockdown/knockout of PBLD increased the mRNA expression of HAdV‐4 Penton base (Pb) and BoHV‐1 gE, respectively (Figure 1C,D), indicating that PBLD deficiency impairs DNA virus‐induced type I IFN responses and facilitates DNA viral replication.

To further investigate the regulatory function of PBLD in cGAS–STING signaling in primary cells. Mouse embryonic fibroblasts (MEFs), peritoneal macrophages (PMs), and bone marrow‐derived macrophages (BMDMs) were isolated from both *Pbld*‐deficient (*Pbld^−/−^)* and WT (*Pbld* ^+/+^) mice. Upon stimulation with HAdV‐4 or ISD, *Pbld* deficiency in MEFs markedly decreased the expression of *Ifna4*, *Ifnb*, and ISGs (*Isg15, Ifitm3*, and *Mx1*) (Figure 1E; Figure S1B, Supporting Information). Moreover, the expression of HAdV‐4 viral gene was upregulated in *Pbld*‐deficient MEFs compared to that in WT controls (Figure 1F). Similar impaired type I IFN responses and increased viral gene expression were observed in HAdV‐4‐infected PMs and BMDMs derived from *Pbld* ^−/^^−^ mice (Figure 1G–J). Collectively, these findings suggest that PBLD promotes cGAS–STING signaling in both immortalized and primary cells.

### 2.2 *Pbld^−/−^* Mice are Highly Susceptible to HAdV‐4 Infection

**Figure 2:**
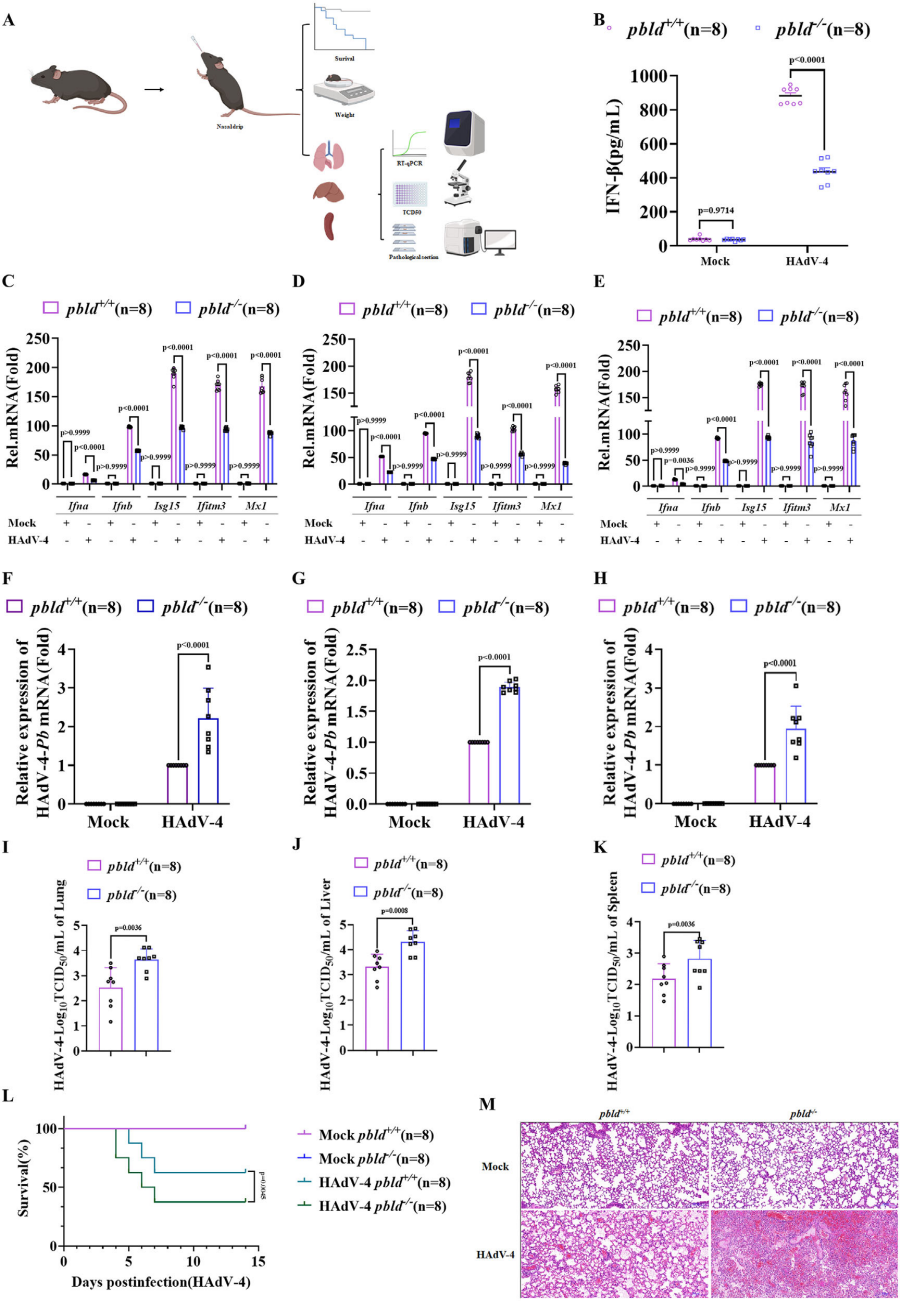
*Pbld*‐deficient mice exhibit increased susceptibility to HAdV‐4 infection. A) A schematic summarizing the workflow of experiments. B) ELISA analysis of IFN‐β production in serum from *Pbld^+/+^* and *Pbld^−/−^* mice intranasally infected with HAdV‐4 (1 × 10^7^ PFU) for 24 h (n = 8 per group). C–E) Real‐time PCR analysis of mRNA expression of IFNs and ISGs in the lungs (C), livers (D), and spleens (E) of *Pbld*⁺^/^⁺ and *Pbld* ^−^^/^^−^ mice 24 h post‐infection with HAdV‐4. F–H) RT‐qPCR analysis of HAdV‐4 *Pb* gene in the lungs (F), livers (G), and spleens (H) from infected *Pbld^+/+^* or *Pbld^−/_^* mice (n = 8 per group). I–K) Viral titers (TCID_50_) of HAdV‐4 in the lungs (I), livers (J), and spleens (K) from *Pbld^+/+^* or *Pbld^−/^* mice (n = 8 per group). L) Survival curves of *Pbld*⁺^/^⁺ and *Pbld* ^−^^/^^−^ mice after HAdV‐4 infection was estimated by Kaplan–Meier method and compared by two‐side log‐rank test (n = 8 for each group). M) Representative H&E staining of lung tissue sections from *Pbld^+/+^* or *Pbld^−/−^* mice with or without HAdV‐4 infection, respectively (n = 8 per group). Scale bar, 100 µm. Data in (B‐K) are presented as mean ± S.D., data in (B‐E) were analyzed by two‐way ANOVA, and data in (F‐K) by t‐test; n = 8, with each data point representing an independent biological experiment. Data in (M) are representative from three independent experiments.

Next, we investigated the physiological and pathological roles of *Pbld* in antiviral immunity in vivo. We intranasally infected *Pbld^+/+^* and *Pbld* ^−/−^ mice with HAdV‐4, followed by comprehensive analyses (**Figure** **2**A). Upon infection with HAdV‐4, *Pbld*‐deficient mice produced lower levels of IFN‐β in sera compared to those in WT mice (Figure 2B). The transcription of *Ifna4, Ifnb*, and ISGs (*Isg15, Ifitm3*, and *Mx1*) in HAdV‐4‐infected lungs, livers, and spleens of *Pbld* ^−/−^ mice were markedly attenuated compared to WT counterparts (Figure 2C–E). In contrast, viral load in the lungs, livers, and spleens of *Pbld* ^−/−^ mice was considerably higher than that in *Pbld* ^+/+^ mice (Figure 2F–H). Consistently, the tissue culture infectious dose 50 (TCID_50_) of HAdV‐4 was significantly elevated in the lungs, livers, and spleens of *Pbld* ^−/−^ mice compared to control mice (Figure 2I–K). Furthermore, *Pbld*‐deficient mice exhibited markedly higher susceptibility to HAdV‐4‐induced lethality than control mice (Figure 2L). Notably, hematoxylin‐eosin (H&E) staining of the alveoli, bronchioles, and trachea of HAdV‐4‐infected *Pbld* ^−/−^ mice exhibited worse tissue damage and inflammatory cell infiltration (Figure 2M). Taken together, these results suggest that PBLD is essential for host defense against DNA viruses in vivo.

### 2.3 PBLD Stabilizes STING by Inhibiting CCDC50‐Dependent Autophagic Degradation

**Figure 3:**
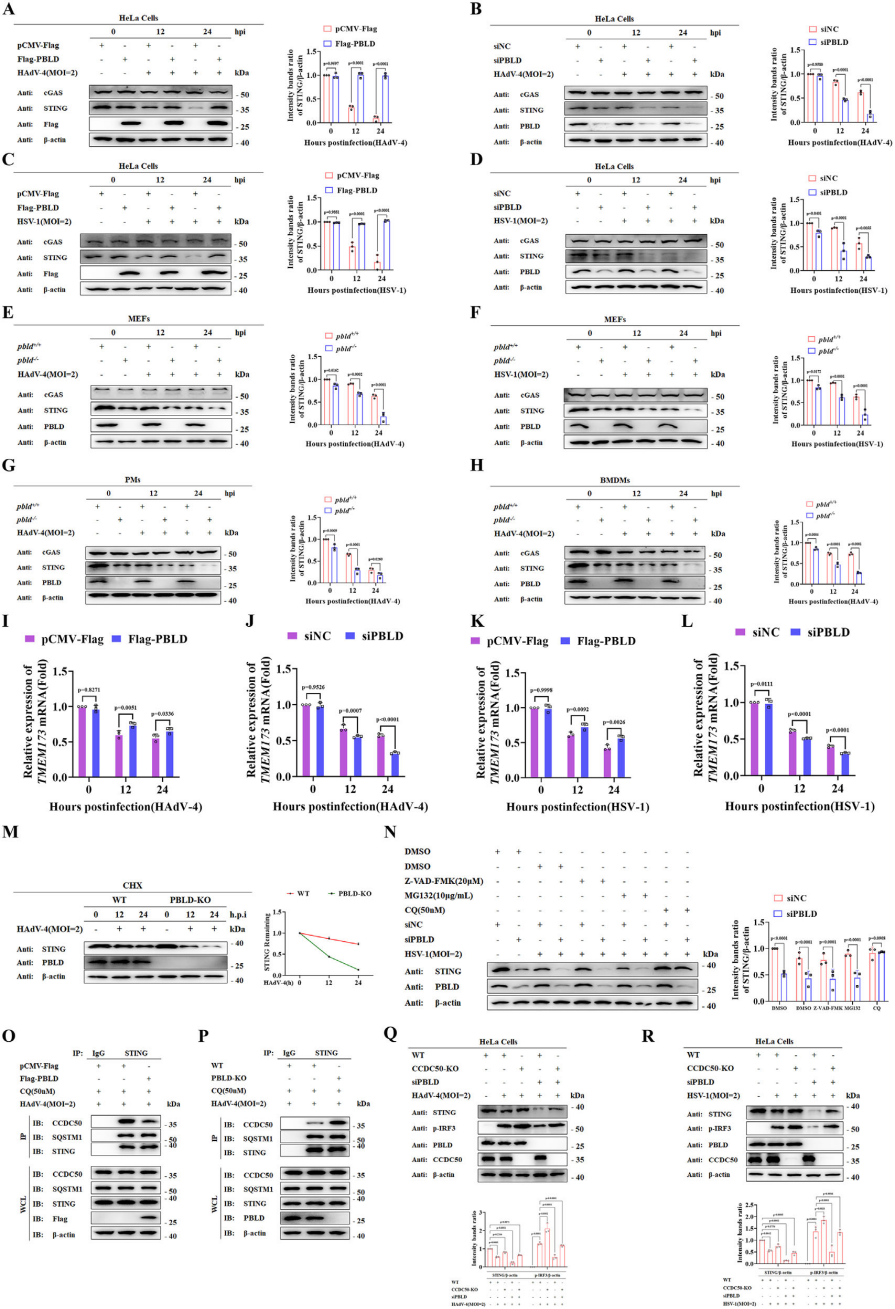
PBLD regulates the cGAS–STING signaling pathway by suppressing STING autophagic degradation. A–D) Immunoblot analysis of specified proteins in PBLD‐overexpressing and PBLD‐silenced HeLa cells infected with HAdV‐4 (MOI = 2) (A, B) or HSV‐1 (MOI = 2) (C,D) for the indicated time points. E,F) Immunoblot analysis of cGAS and STING in MEFs from *Pbld^+/+^* or *Pbld^−/−^* mice infected with HAdV‐4 (E) or HSV‐1 (F) over time. G,H) Immunoblot analysis of cGAS and STING in PMs (G) and BMDMs (H) from *Pbld^+/+^* or *Pbld^−/−^* mice infected with HAdV‐4. I–L) RT‐PCR analysis of *STING* (*TMEM173*) mRNA expression in PBLD‐overexpressing (I, K) and PBLD‐silenced (J, L) HeLa cells during HAdV‐4 or HSV‐1 infection. M) Western blotting analysis of STING protein levels in HSV‐1‐infected control or PBLD‐KO HeLa cells treated with cycloheximide (CHX, 100 ng mL^−1^). The STING intensity was quantified and normalized to β‐actin. N) Western blotting analysis of specified proteins expression in HSV‐1‐infected control or PBLD‐silenced HeLa cells treated with Z‐VAD‐FMK (20 µm), MG132 (10 µg mL^−1^) or CQ (50 nm) for 12 h. O,P) HeLa cells transfected with Flag‐PBLD or control vector (O) or wild type and PBLD knockout HeLa cell lines (P) were treated with CQ (50 nm) and infected with HAdV‐4(MOI = 2) for 12 h. Cells lysates were immunoprecipitated with anti‐STING and immunoblotted with specified protein antibodies. Q,R) Wild‐type and CCDC50‐KO cell lines transfected with siPBLD (10 nm) or scrambled siRNA (siNC) were infected with HAdV‐4(MOI = 2) (Q) or HSV‐1(MOI = 2) (R) for 12 h, followed by immunoblot analysis of indicated proteins. Data in (A‐H, M‐R) are representative of three independent experiments. Band intensities in (A‐H, M, N, Q, R) were quantified using ImageJ and are presented as mean ± S.D. Data in (I‐L) and band intensity quantifications were analyzed by two‐way ANOVA; n  =  3.

To elucidate the molecular mechanism by which PBLD positively regulates the cGAS–STING pathway, we first examined its impact on the stability of cGAS and STING. As shown in **Figure** **3**A, PBLD overexpression during HAdV‐4 infection upregulated STING expression without affecting cGAS expression compared to the control vector, whereas PBLD knockdown produced the opposite effects (Figure 3B). Similarly, PBLD overexpression or knockdown upregulated or downregulated STING protein expression, respectively, in response to herpes simplex virus type 1 (HSV‐1) infection (Figure 3C,D). Consistent findings were observed in *Pbld* deficient MEFs infected with HAdV‐4 or HSV‐1, as well as in PMs and BMDMs infected with HAdV‐4 (Figure 3E–H). Although RT‐qPCR analysis revealed PBLD‐dependent modulation of *TMEM173 (STING)* mRNA levels following HAdV‐4 or HSV‐1 infection (Figure 3I–L), cycloheximide (CHX) chase assays showed that the degradation rates of STING was increased in PBLD‐KO cells compared with that in WT cells, suggesting that PBLD stabilizes the STING protein (Figure 3M). These results indicate that PBLD regulates STING at both the mRNA and protein levels. Given the established role of STING turnover in regulating cGAS–STING signaling dynamics, we further investigated how PBLD controls STING protein stability using pharmacological protein inhibitors. Pharmacological blockade experiments showed that STING degradation in the absence of PBLD was effectively prevented by the autophagy inhibitor chloroquine (CQ), but not by the ubiquitin‐proteasome inhibitor MG132 or the caspase inhibitor Z‐VAD‐FMK (Figure 3N). Additionally, immunofluorescence assays demonstrated that PBLD knockout enhanced STING localization to lysosomes (Figure S2A, Supporting Information), indicating that PBLD deficiency predominantly promotes STING degradation through the autolysosomal pathway. We further examined the role of PBLD in autophagy‐related 7 knockout (ATG7‐KO) HeLa cells, in which autophagy is impaired. STING protein levels in these cells remained stable in the absence of PBLD (Figure S2B, Supporting Information), demonstrating that PBLD inhibits the autophagic degradation of STING.

Recent evidence has demonstrated the critical regulatory function of cargo receptors in orchestrating selective autophagic degradation. Within the cGAS–STING pathway, several autophagic adaptors—including SQSTM1/p62 and CCDC50—have been shown to mediate STING turnover.^[^ ^18^, ^19^, ^20^ ^]^ To determine whether PBLD regulates STING degradation through these cargo receptors, we performed co‐immunoprecipitation(co‐IP) analysis and found that PBLD overexpression significantly disrupted the association between STING and the autophagic receptor CCDC50, but not SQSTM1/P62 (Figure 3O). Conversely, PBLD knockout exerted the opposite effects (Figure 3P). Notably, the co‐localization of CCDC50 and STING was increased in PBLD‐KO cells compared with that in control cells following HAdV‐4 infection (Figure S2C, Supporting Information). In addition, CCDC50‐KO blocked STING degradation and enhanced IRF3 phosphorylation in HAdV‐4– and HSV‐1– infected PBLD knockdown cells (Figure 3Q,R). Taken together, these results indicate that PBLD stabilizes STING by functionally antagonizing CCDC50‐mediated autophagic degradation.

### 2.4 PBLD Attenuates K48‐Linked Polyubiquitination of STING at K150

**Figure 4:**
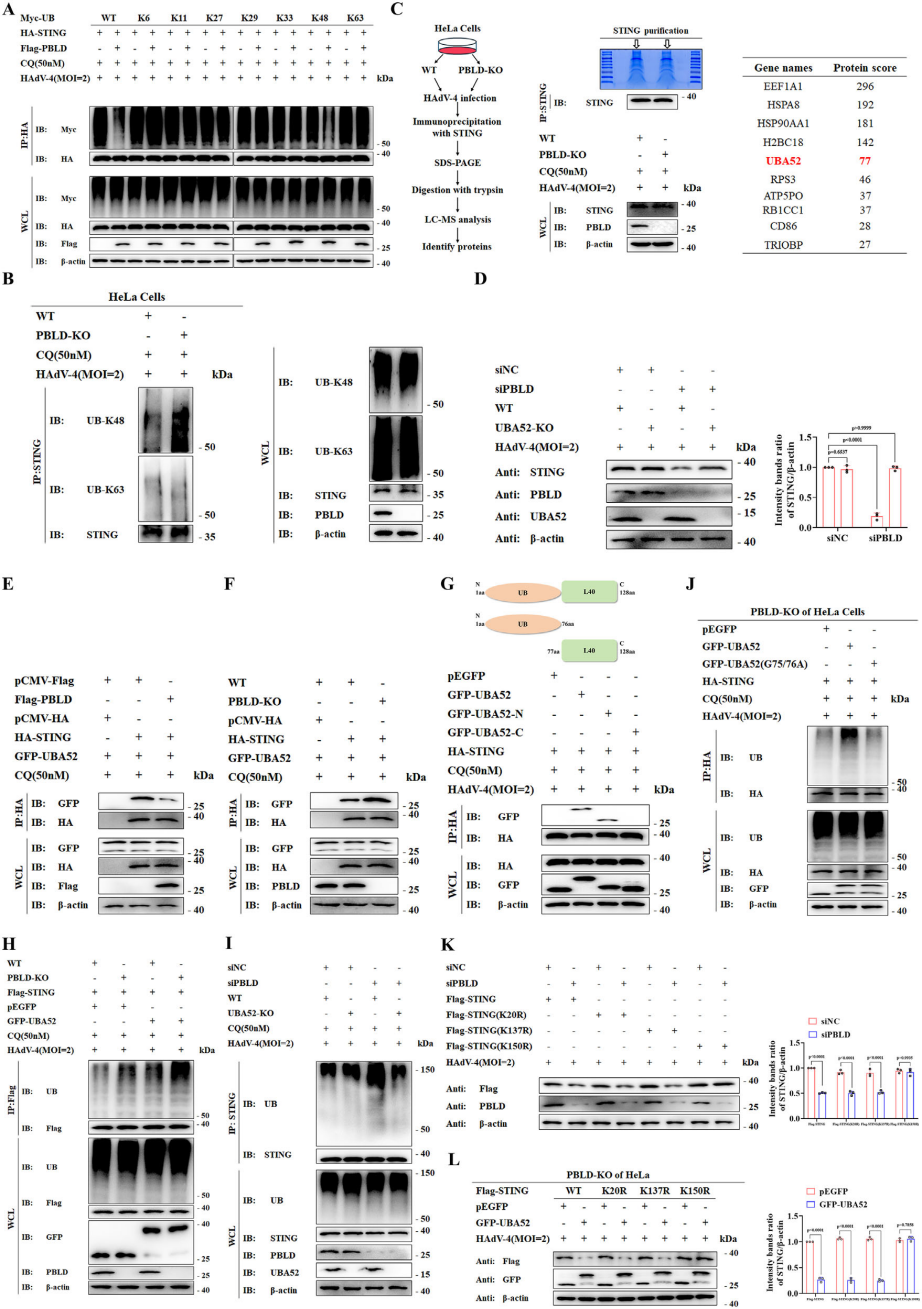
PBLD suppresses K48‐linked ubiquitination of STING at K150 to prevent its autophagic degradation. A) Co‐IP analysis of STING ubiquitination in HeLa cells transfected with indicated plasmids and Myc‐Ub (wild‐type and the indicated mutants). B) Co‐IP analysis of endogenous K48‐ and K63‐linked ubiquitination of STING in PBLD‐KO and wild‐type (WT) cell lines treated with CQ (50 nm) and infected with HAdV‐4 (MOI = 2) for 12 h. C) Scheme of working model for STING‐interacting proteins identified by mass spectrometry in PBLD‐KO and WT cell lines infected with HAdV‐4(MOI = 2) for 12 h. D) Immunoblot analysis of indicated proteins in WT and UBA52‐KO HeLa cells transfected with siPBLD or control siRNA (siNC) and infected with HAdV‐4. E,F) Co‐IP analysis in HeLa cells (E) or WT and PBLD‐KO HeLa cells (F), transfected as indicated. Lysates were immunoprecipitated with anti‐HA beads and immunoblotted with anti‐GFP antibody. G) Schematic diagram of truncated UBA52 (Above). Co‐IP analysis in HeLa cells co‐transfected with indicated vectors, treated with CQ, and infected with HAdV‐4 (Below). H,I) Co‐IP analysis of STING polyubiquitination in WT and PBLD‐KO cells transfected as indicated (H), or in WT and UBA52‐KO cells transfected with siPBLD or siNC (I), followed by CQ (50 nm) treatment and HAdV‐4 (MOI = 2) infection. J) Co‐IP analysis of STING polyubiquitination in PBLD‐KO HeLa cells co‐transfected with HA‐STING and pEGFP, GFP‐UBA52, or GFP‐UBA52(G75/76A) mutant, and infected with HAdV‐4(MOI = 2) in the presence of CQ (50 nm). K,L) Immunoblot analysis of indicated proteins in WT and PBLD‐KO HeLa cells (K) or PBLD‐KO HeLa cells (L) transfected with indicated vectors and infected with HAdV‐4. Data in (A, B, D‐L) are representative from three independent experiments. The gray intensity of the bands in data (D, K, L) was quantified from three independent experiments using ImageJ software and are presented as mean ± S.D., two‐way ANOVA.

Ubiquitin chains conjugated to the substrates are well established as signals for the recognition by cargo receptors.^[^ ^21^, ^22^, ^23^ ^]^ We hypothesized that PBLD may affect STING ubiquitination, thereby influencing its subsequent CCDC50‐dependent degradation. To test this, we examined the specific ubiquitin linkage types regulated by PBLD. Overexpression of PBLD specifically decreased both total and K48‐linked ubiquitination (K48‐only ubiquitin mutant) of STING, but did not affect other ubiquitin linkages (K6‐, K11‐, K27‐, K29‐, K33‐ or K63‐only ubiquitin mutants) (**Figure** **4**A). In contrast, PBLD‐KO increased K48‐linked polyubiquitination of STING, but not K63‐linked modification (Figure 4B). Notably, viral infection increased K48‐linked STING ubiquitylation, and this effect was further enhanced in PBLD‐deficient cells (Figure S3A, Supporting Information). Given that PBLD is not an E3 ubiquitin ligase, and coimmunoprecipitation assays demonstrated that PBLD did not interact with STING, and vice versa (Figure S3B,C, Supporting Information), we hypothesized that specific additional molecules are likely to mediate STING ubiquitination. To address this, we performed immunoprecipitation combined with mass spectrometry analysis in PBLD‐KO and control cell lines and identified 10 potential cellular interactors of STING by applying a total score threshold of >25. Among these, we selected ubiquitin A‐52 residue ribosomal protein fusion product 1 (UBA52), owing to its close association with ubiquitin signaling, which may influence STING degradation via autophagy upon PBLD knockout (Figure 4C). Moreover, UBA52‐KO restored STING protein levels in PBLD‐silenced cells during HSV‐1 infection (Figure 4D). Furthermore, PBLD overexpression attenuated the interaction between STING and UBA52 (Figure 4E), whereas PBLD‐KO produced the opposite effect (Figure 4F), suggesting that PBLD negatively regulates the autophagic degradation of STING by suppressing the recruitment of UBA52 to STING.

To further investigate the regulatory role of PBLD in UBA52‐driven ubiquitination and degradation of STING, we generated UBA52 truncations containing either the N‐terminal ubiquitin domain or the C‐terminal ribosomal protein L40 (RPL40). Co‐IP assays revealed that the N‐terminal ubiquitin domain of UBA52 specifically bound STING, whereas the C‐terminal RPL40 domain did not (Figure 4G), suggesting that ubiquitin derived from UBA52 may modify STING. As expected, PBLD knockout or knockdown substantially increased STING ubiquitination upon UBA52 overexpression (Figure 4H), whereas UBA52 deficiency reduced this effect (Figure 4I). To confirm that ubiquitin was derived from the cleavage of UBA52, we constructed a cleavage‐resistant (CR) UBA52 expression vector with glycine (G)‐to‐alanine (A) substitutions at positions 75/76 within the ubiquitin C‐terminus (GFP‐UBA52^G75/76A^). Notably, PBLD knockout enhanced STING ubiquitination in cells expressing WT UBA52 but not in those expressing the CR mutant (Figure 4J), confirming that ubiquitin released from UBA52 proteolytic processing is essential for STING modification.

Next, we employed the UbPred algorithm (http://www.ubpred.org) to predict potential ubiquitination sites on STING and generated STING mutants with lysine (K)‐to‐arginine (R) substitutions at three potential ubiquitination sites (K20, K137, and K150). In PBLD‐deficient HeLa cells, mutation of K150, but not K20 or K137, abolished PBLD knockdown‐induced STING degradation (Figure 4K). Notably, UBA52 further augmented the degradation of WT STING and the STING^K20R/K137R^ mutants, but not the STING^K150R^ mutant, in HAdV‐4‐infected PBLD‐KO cell lines (Figure 4L), suggesting that the lysine residue at position 150 (K150) of STING is required for its K48‐linked ubiquitination. Taken together, PBLD inhibits autophagic degradation of STING by suppressing UBA52‐driven K48‐linked ubiquitination of STING at K150.

### 2.5 PBLD Promotes STING‐Mediated Immune Responses by Attenuating CCDC50‐ Recognized Degradation of STING

**Figure 5:**
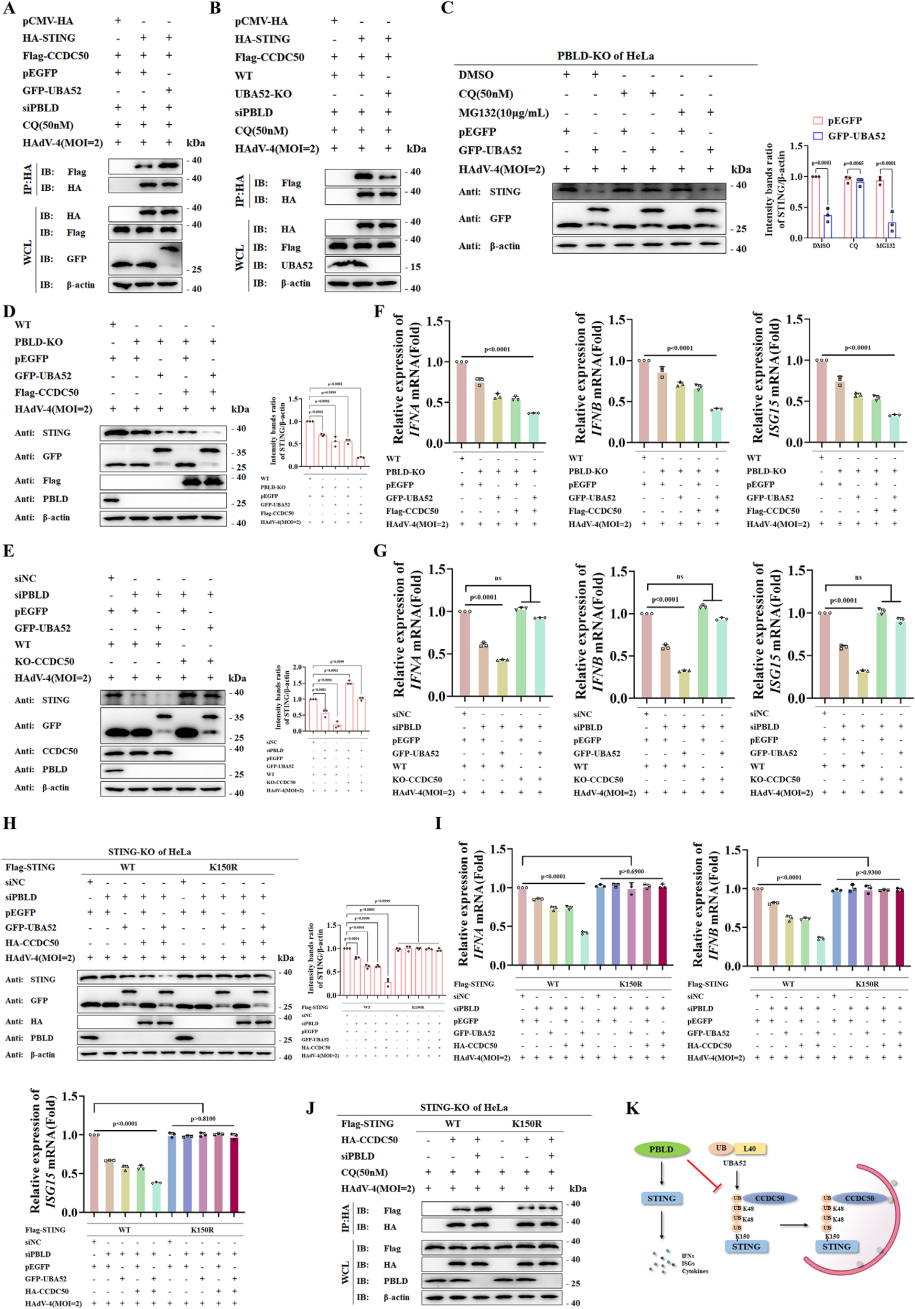
PBLD suppresses CCDC50‐mediated STING autophagic degradation to enhance the IFN‐I response. A,B) Co‐IP analysis of the STING‐CCDC50 interaction in PBLD‐silenced HeLa cells transfected with indicated vectors (A), or in WT and UBA52‐KO HeLa cells transfected with siPBLD and indicated vectors (B), following CQ treatment and HAdV‐4 infection. C) Immunoblot analysis in PBLD‐KO HeLa cells transfected with pEGFP or GFP‐UBA52 and treated with DMSO, MG132, or CQ during HAdV‐4 infection. D,E) Immunoblot analysis in WT and PBLD‐KO HeLa cell lines (D) or WT and CCDC50‐KO HeLa cells (E) co‐transfected with indicated expression vectors or siPBLD and infected with HAdV‐4(MOI = 2) for 12 h. F,G) RT‐qPCR analysis of *IFNA*, *IFNB*, and *ISG15* expression in WT and PBLD‐KO HeLa cell lines (F) or WT and CCDC50‐KO HeLa cells (G) co‐transfected with indicated vectors or siPBLD and infected with HAdV‐4 (MOI = 2) for 12 h. H,I) Immunoblotanalysis of indicated proteins (H) and RT‐qPCR analysis of IFNs and ISGs expression (I) in STING‐KO HeLa cells transfected with siPBLD, GFP‐UBA52, and HA‐CCDC50, along with either WT STING or the STING (K150R) mutant, followed by HAdV‐4 (MOI = 2) infection. J) Co‐IP analysis in STING‐KO HeLa cells transfected with HA‐CCDC50 and siPBLD, along with either WT STING or STING (K150R) mutant, followed by HAdV‐4 (MOI = 2) infection. K) A proposed model illustrating how the PBLD–STING–CCDC50 cargo receptors axis negatively regulates STING‐mediated antiviral immune responses. Data in (F, G, I) are presented as mean ± S.D., two‐way ANOVA; n = 3 biological independent experiments. Data in (A‐E, H, J) are representative from three independent experiments. Band intensities in data (C‐E, H) was quantified from three independent experiments using ImageJ software, and analyzed by one‐way ANOVA for data (D, E), two‐way ANOVA for data (C, H).

Based on the observation that CCDC50 knockout abolished PBLD silencing‐induced STING degradation (Figure 3Q,R), we speculated that UBA52‐mediated ubiquitination might provide a recognition signal for CCDC50. To test this, we analyzed the effect of UBA52 on the interaction between STING and CCDC50 was analyzed. Co‐IP in PBLD‐ silenced cells revealed that overexpression of UBA52 enhanced STING binding to CCDC50 (**Figure** **5**A), whereas UBA52 KO reduced this interaction (Figure 5B). Furthermore, in PBLD‐deficient cells, the increased degradation of STING by UBA52 overexpression was reversed by the autophagy inhibitor CQ, but not by the proteasome inhibitors MG132 (Figure 5C), indicating that UBA52 plays a critical role in STING autophagic degradation. Next, we explored the role of CCDC50 in UBA52‐mediated STING degradation. UBA52 overexpression in PBLD‐silenced cells further promoted STING degradation (Figure 5D), whereas this effect was abolished upon CCDC50 knockout (Figure 5E). Furthermore, we examined whether CCDC50‐mediated STING degradation is required for antiviral immunity. As shown in Figure 5F, UBA52 overexpression, either alone or in combination with CCDC50, downregulated type I IFN and ISGs expression. However, this effect was not observed in cells in which CCDC50 was knocked out (Figure 5G). These results suggest that PBLD promotes cGAS–STING signaling activation by inhibiting CCDC50‐mediated autophagic degradation of STING.

Subsequently, using STING‐KO HeLa cell lines reconstituted with either WT STING or mutant STING (STING^K150R^), we observed that UBA52 and CCDC50 synergistically promoted WT STING degradation, but not that of the STING^K150R^ mutant upon PBLD silencing (Figure 5H), suggesting that PBLD targets and stabilizes STING in a K150‐dependent manner. Functional analyses further revealed that, in the presence of UBA52 and CCDC50, the STING K150R mutant failed to affect HAdV4‐induced type I IFN responses compared to WT STING (Figure 5I). Consistently, knockout of PBLD enhanced the interaction of WT STING and CCDC50 but had minimal effect on the STING^K150R^ mutant (Figure 5J). Collectively, these findings indicate that PBLD potently suppresses UBA52‐driven K48‐linked ubiquitination of STING at K150, thereby preventing STING degradation and ultimately sustaining STING‐mediated immune responses (Figure 5K).

### 2.6 Viral Infection Downregulates PBLD Expression Through TFEB and MARCH2

**Figure 6:**
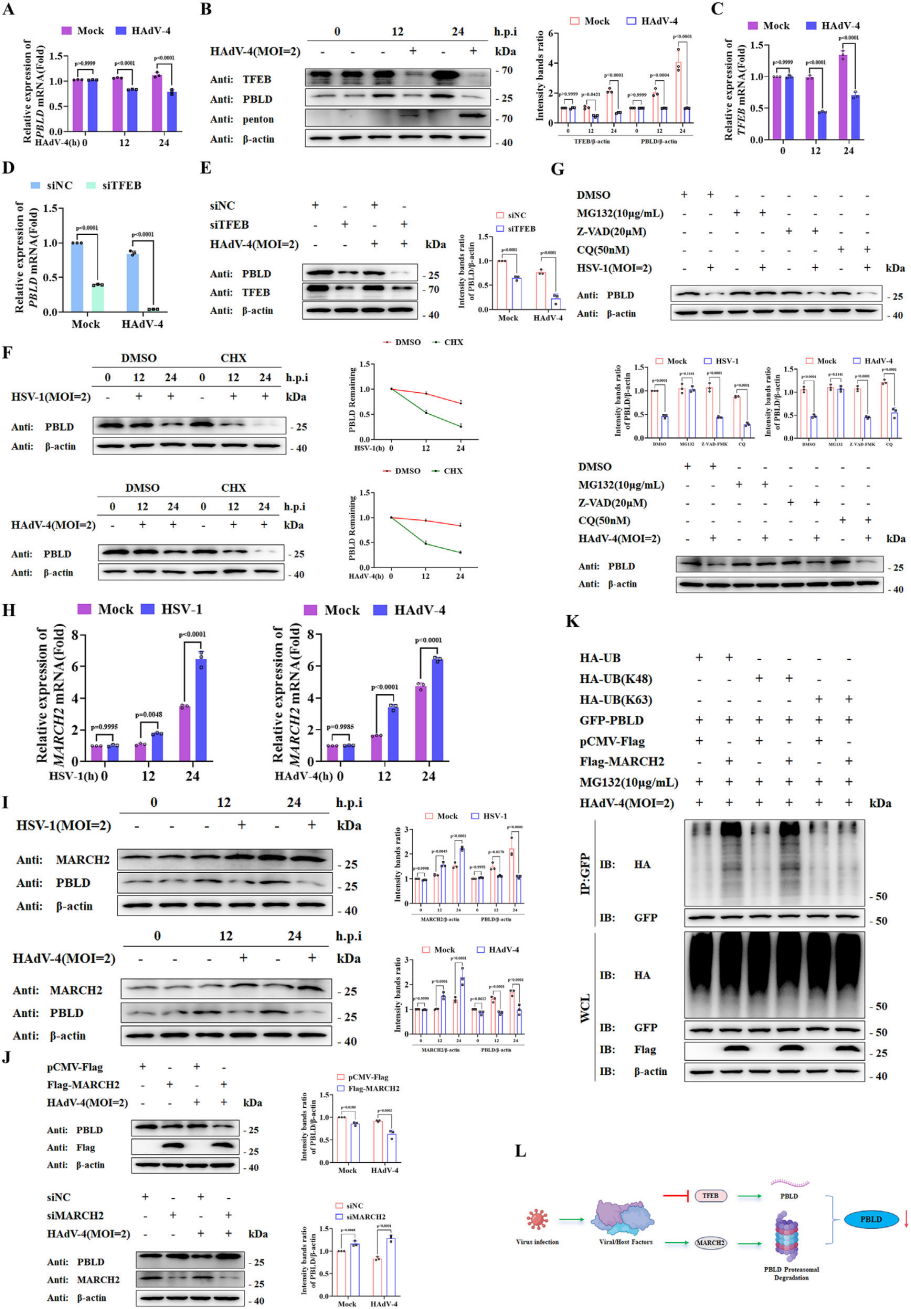
Viral infection downregulates PBLD through TFEB and MARCH2. A,C) RT‐PCR analysis of *PBLD* (A) and *TFEB* (C) expression in HAdV‐4 infected HeLa cells for the indicated time points. B) Western blot analysis of the indicated protein following HAdV‐4 infection as the indicated MOI and time points. D,E) RT‐qPCR analysis of *PBLD* mRNA (D) and immunoblot analysis for indicated protein in HeLa cells (E) transfected with siTFEB (10 nm) or scrambled siRNA (siNC), followed by mock infection or HAdV‐4(MOI = 2) infection for 12 h. F) Western blotting analysis of PBLD protein levels in HeLa cells treated with DMSO (vehicle control) or CHX (100 ng mL^−1^) followed by HSV‐1 or HAdV‐4 infection for the indicated times. The STING intensity was quantified and normalized to β‐actin. G) Western blotting analysis of PBLD protein levels in HeLa cells that were uninfected or infected with HSV‐ 1 (MOI = 2) or HAdV‐4 (MOI = 2) and then treated with protein degradation inhibitors for 12 h. H,I) RT‐PCR and Western blot analysis of MARCH2 expression in HSV‐1‐ or HAdV‐4‐infected HeLa cells for the indicated time points. J) Western blotting analysis of PBLD protein level in HeLa cells transfected with MARCH2 overexpression or siMARCH2 (10 nm) or scrambled siRNA (siNC) and then treated with mock infection or HAdV‐4(MOI = 2) infection for 12 h. K) The HeLa cells were transfected with indicated vectors for 36 h and treated with MG132 followed by co‐IP analysis. L) Schematic diagram of the mechanism that viral infection downregulates PBLD via downregulation of TFEB and upregulation of MARCH2. Data in (A, C, D, G) are presented as mean ± S.D., two‐way ANOVA; n = 3 biological independent experiments. Data in (B, E‐G, I–K) are representative of three independent experiments. The gray intensity of the bands in data (B, E, F, G, I, J) were analyzed from three independent experiments using ImageJ software and presented as mean ± S.D., two‐way ANOVA.

Our previous studies have demonstrated that certain RNA viral infections downregulate PBLD expression.^[^ ^15^, ^17^ ^]^ To extend these findings, we investigated the effect of DNA viral infection on PBLD expression. HSV‐1 and HAdV‐4 infections downregulated PBLD expression at both mRNA and protein levels (**Figure** **6A,B**; Figure S4A,B, Supporting Information). Given the established role of TFEB in modulating PBLD transcription.^[^ ^24^ ^]^ we examine the TFEB dynamics during DNA viral infection. Notably, both HSV‐1 and HAdV‐4 infections suppressed TFEB expression at the mRNA and protein levels (Figure 6B,C; Figure S4B,C, Supporting Information). Furthermore, TFEB knockdown downregulated PBLD mRNA and protein expression regardless of viral infection, whereas TFEB overexpression exerted the opposite effects (Figure 6D,E; Figure S4D,E, Supporting Information). These findings suggest that TFEB‐mediated transcriptional regulation of PBLD represents a common mechanism that viral infections further downregulate TFEB expression, thereby suppressing PBLD transcription.

PBLD protein levels in DNA virus‐infected cells were also significantly reduced (Figure 6B). CHX chase assays revealed enhanced PBLD degradation in HeLa cells upon HSV‐1 or HAdV‐4 infection (Figure 6F). To determine the primary degradation pathway responsible for this effect, we assessed PBLD protein levels using inhibitors targeting different degradation pathways. Western blot analysis demonstrated that the ubiquitin–proteasome inhibitors MG132, but not the autophagy (CQ) and the caspase inhibitors (Z‐VAD‐FMK), restored PBLD levels following HAdV‐4 or HSV‐1 infection (Figure 6G), implicating the ubiquitin–proteasome pathway in this process. Previous studies have reported that the E3 ligase MARCH2 acts as a negative regulator of MAVS‐ and NF‐κB‐mediated signaling during viral infection.^[^ ^25^, ^26^ ^]^ It is negatively correlated with the positive regulatory role of PBLD in MAVS‐ and NF‐κB‐mediated innate immune responses. We therefore hypothesized that MARCH2 may be involved in regulating PBLD expression. Subsequently, the interaction between MARCH2 and PBLD was predicted by molecular docking (Figure S4F, Supporting Information). DNA viral infection markedly upregulated MARCH2 expression at both mRNA and protein levels (Figure 6H,I). Furthermore, MRACH2 overexpression resulted in decreased PBLD protein expression, whereas siRNA‐mediated MARCH2 knockdown produced the opposite effect (Figure 6J). To elucidate the mechanistic relationship between MARCH2 and PBLD, we investigated the role of MARCH2 in regulating PBLD stability. DNA viral infection promoted K48‐linked ubiquitination of PBLD, which was further enhanced by MARCH2 overexpression, but not the K63‐linked ubiquitination (Figure 6K), indicating that MARCH2 may be recruited to promote the K48‐linked PBLD polyubiquitination and degradation of PBLD. Consistently, RNA viral infection downregulated TFEB and upregulated MARCH2 expression at both the transcriptional and protein levels (Figure S4G–I, Supporting Information). These findings indicate that both DNA and RNA viral infections downregulate PBLD transcription by suppressing TFEB expression, while simultaneously promoting PBLD degradation via upregulation of the MARCH2‐mediated ubiquitin‐proteasome pathway (Figure 6L).

### 2.7 PBLD Deficiency Alleviates Autoimmunity in a TMPD‐Induced Murine Lupus Model

**Figure 7:**
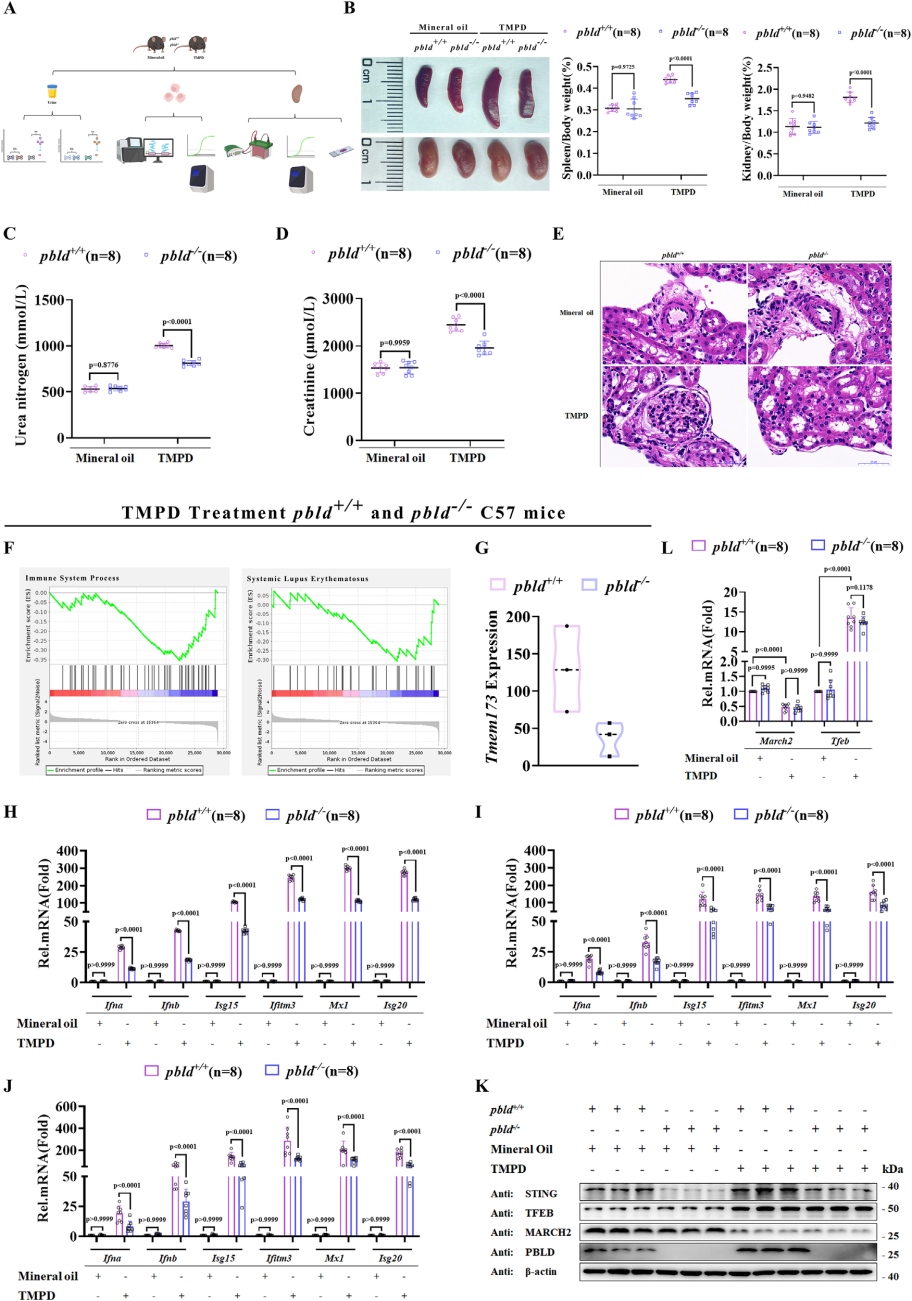
PBLD deficiency alleviates autoimmunity in a TMPD‐induced murine lupus model. A) Schematic diagram of the TMPD‐induced lupus model for related detection. B) Representative images of spleen and spleen weight/body weight ratios, kidney and kidney weight/body weight ratios from *Pbld* ^+/+^, *Pbld* ^−/−^mice induced by TMPD (0.5 mL per mouse) or Mineral (vehicle control). C,D) Quantitative analysis of urea nitrogen (C) and creatinine (D) in urine samples from mineral oil or TMPD (0.5 mL per mouse)‐treated *Pbld* ^+/+^ and *Pbld* ^−/−^ mice. E) Representative images of H&E‐stained kidney sections from *Pbld* ^+/+^, *Pbld* ^−/−^mice induced by TMPD or Mineral (scale bar 100 µm). F) GSEA snapshots of KEGG pathway enrichment analysis: Immune System Process and SLE (*Pbld* ^−/−^ vs *Pbld* ^+/+^). G) RNA‐Seq analysis of the mRNA expression of *Tmem173* in BMDMs from *Pbld* ^+/+^ and *Pbld* ^−/−^mice after TMPD treatment. H–J) Real‐time PCR analysis of IFNs and ISGs mRNA levels in PMs (H), BMDMs (I), and kidneys (J) from mineral oil or TMPD‐treated *Pbld* ^+/+^ and *Pbld* ^−/−^ mice. K) Immunoblot analysis the indicated proteins in kidneys from mineral oil or TMPD‐treated *Pbld* ^+/+^ and *Pbld* ^−/−^ mice. Data in (B right, C, D, H–J, K) are presented as mean ± S.D., two‐way ANOVA; n = 8 biological independent experiments. Data in (B left, E, K) are representative from three independent experiments. The box plots in (G) are defined in terms of minima, maxima, centre, bounds of box and whiskers, and percentile. Each symbol represents an individual mouse.

Accumulating evidence has revealed that the aberrant activation of the cGAS–STING signaling pathway plays a critical pathogenic role in SLE.^[^ ^27^ ^]^ Considering the prominent role of PBLD in regulating STING‐mediated type I IFN signaling, we sought to determine whether PBLD is involved in SLE pathogenesis. To investigate the potential functions of PBLD in SLE, we established the TMPD‐induced murine lupus model, one of the most commonly used murine lupus models.^[^ ^28^, ^29^ ^]^ Six‐week‐old female *Pbld* ^+/+^ and *Pbld* ^−/−^ mice were injected with TMPD, and disease progression was monitored (**Figure** **7**A). Consistent with the hallmark systemic inflammation of SLE, which affects multiple organs, TMPD‐treated *Pbld* ^+/+^ mice developed more severe inflammatory responses manifested as splenic and renal swelling and increased organ weight, compared with *Pbld* ^−/−^ mice (Figure 7B). Lupus nephritis is a key pathological feature of SLE, characterized by glomerular immune complex deposition and accumulation of urea nitrogen and creatinine.^[^ ^29^, ^30^ ^]^ Following TMPD treatment, control mice exhibited significantly higher levels of creatinine and urea nitrogen than *Pbld* ^−/−^ mice (Figure 7C,D). H&E‐stained kidney sections showed that TMPD‐treated *Pbld* ^+/+^ mice developed more severe inflammatory cell infiltration than *Pbld^−/−^* mice (Figure 7E). Furthermore, RNA sequencing (RNA‐seq) analysis of TCGA datasets of BMDMs from TMPD‐treated *Pbld* ^+/+^ and *Pbld^−/−^* mice indicated a significant association between PBLD expression and SLE‐relevant pathways, particularly those related to immune system processes (Figure 7F). Consistently, RNA‐seq data indicated that TMPD administration in *Pbld* ^+/+^ mice increased *Tmem173* mRNA expression compared with that in *Pbld^−/−^* mice (Figure 7G), suggesting that PBLD deficiency in the TMPD‐induced murine lupus mice model leads to attenuated autoimmune phenotypes

To investigate type I IFN pathway activation in TMPD‐induced SLE mice, we analyzed PMs and BMDMs and demonstrated that both PMs and BMDMs from *Pbld^−/−^* mice exhibited impaired TMPD‐induced expression of *Ifna, Ifnb, Isg15, Ifitm3, Mx1*, and *Isg20*, compared with WT controls (Figure 7H,I). Consistent with these findings, TMPD administration induced higher expression of these mRNA in the kidneys of *Pbld* ^+/+^ mice than in *Pbld* ^−/−^ mice (Figure 7J), consistent with the positive role of PBLD in regulating the expression of these genes. Notably, STING expression was significantly increased in the kidneys of the *Pbld^+/+^* group compared with that in the *Pbld* ^−/−^ control group (Figure 7K). Moreover, reduced STING ubiquitination was observed in TMPD‐treated *Pbld^+/+^* mouse kidneys compared with that in mineral oil‐treated controls (Figure S5, Supporting Information). Given that PBLD expression is regulated by TFEB and MARCH2, we examined their expression and found that TMPD treatment upregulated *Tfeb* and downregulated *March2* (Figure 7L), suggesting that dysregulated TFEB and MARCH2 may modulate PBLD expression in SLE. Collectively, these results demonstrate that *Pbld* deficiency attenuates autoimmune phenotypes and immune responses in the TMPD‐induced lupus model.

### 2.8 PBLD Expression is Upregulated and Positively Correlated with Disease Progression in Patients with SLE

**Figure 8:**
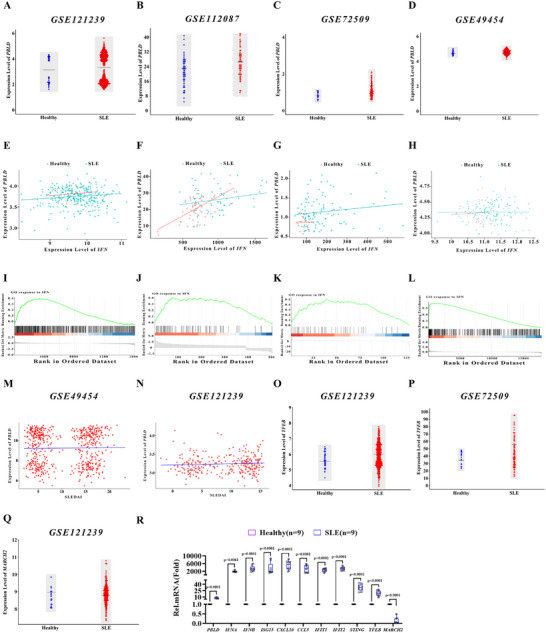
PBLD expression is positively correlated with SLE disease progression. A–D) Comparison of *PBLD* mRNA expression in patients with SLE and healthy donors across four SLE cohorts. One plot represents one GEO dataset. The mean and standard deviation (black lines) are also plotted. E–H) Correlation analysis between the expression levels of PBLD and IFN in patients with SLE and healthy donors across four SLE cohorts. One plot represents one GEO dataset. I–L) GSEA plots showing that PBLD positively correlated with the type I IFN pathway in different GEO datasets. M,N) Correlations between *PBLD* expression and the SLE disease activity index (SLEDAI) in patients from two cohorts. O–Q) Comparison of TFEB (O, P) and MARCH2 (Q) expression in patients with SLE and healthy donors in two SLE cohorts. R) Real‐time qPCR analysis of the indicated gene expression in the blood sample from patients with SLE (n = 9) and healthy donors (n = 9). Data in (R) are presented as mean ± S.D., two‐way ANOVA; n = 9 biological independent experiments.

Considering the prominent role of PBLD in regulating STING‐mediated type I IFN responses in the TMPD‐induced murine lupus model, we investigated its expression patterns in patients with SLE. We performed an integrated analysis of PBLD expression using four independent cohorts from the publicly available Gene Expression Omnibus (GEO) database (GSE121239, GSE112087, GSE72509, and GSE49454). Significant upregulation of *PBLD* mRNA was observed in patients with SLE compared to healthy controls (**Figure** **8**A–D). Notably, PBLD expression was positively correlated with IFN expression, particularly in patients with SLE (Figure 8E–H). Furthermore, PBLD expression levels were positively associated with type I IFN pathway activation in these patients (Figure 8I–L). Subsequently, we reanalyzed two public datasets using the Disease Activity Index (SLEDAI) (GSE49454 and GSE121239). Importantly, *PBLD* mRNA levels were positively correlated with the SLE disease activity index (Figure 8M,N), indicating that PBLD expression is associated with disease severity in SLE.

To further explore whether TFEB regulates PBLD expression in patients with SLE, we performed an integrative analysis using two public datasets (GSE21239 and GSE72509). Notably, *TFEB* mRNA levels were significantly higher in patients with SLE than in healthy donors (Figure 8O,P). Consistently, *MARCH2* mRNA levels were significantly lower in patients with SLE than in healthy donors (Figure 8Q), supporting the hypothesis that dysregulated TFEB and MARCH2 contribute to PBLD upregulation in SLE. Notably, consistent with this regulatory axis, upregulated mRNA levels of *PBLD*, *IFNA, IFNB*, and ISGs (*ISG15, CXCL10, CCL5, IFIT1, IFIT2, STING*, and *TFEB*), alongside downregulated MARCH2 expression, were markedly observed in the clinical blood samples of patients with SLE compared with healthy controls. (Figure 8R). Collectively, these findings indicate that PBLD expression is upregulated and promotes the STING‐mediated pathway, thereby exacerbating aberrant type I IFNs in patients with SLE.

## 3 Discussion

Although the production of IFN and proinflammatory cytokines establishes a critical antiviral state that eliminates intracellular pathogens, aberrant activation of the cGAS–STING pathway can lead to severe autoimmune or autoinflammatory disorders. However, the mechanisms underlying the tight regulation of this pathway remain largely undefined. In this study, we identified PBLD as a positive regulator of dsDNA‐induced type I IFN activation and SLE pathogenesis via the STING signaling axis.

The type I IFN pathway serves as a crucial component of the innate immune defense against pathogenic invasion. Our previous studies have elucidated the multifaceted role of PBLD in orchestrating antiviral immune responses. Mechanistically, PBLD enhances RLR signaling by activating the p53–USP4–MAVS axis in response to RNA virus infections.^[^ ^15^ ^]^ PBLD facilitates virus‐induced type I interferon responses through IRF3‐dependent mechanisms.^[^ ^17^ ^]^ Moreover, PBLD upregulates type I IFN response through activating the NF‐κB signaling pathway.^[^ ^16^ ^]^ Notably, PBLD enhanced antiviral immunity by stabilizing STING expression and attenuating its autophagic degradation. Collectively, these findings establish PBLD as a master regulator that orchestrates innate immune responses to both DNA and RNA viruses through distinct yet complementary mechanisms, underscoring its pivotal role in antiviral immunity and its therapeutic potential.

In this study, we found that PBLD expression was decreased at both the mRNA and protein levels following HSV‐1 and HAdV‐4 stimulation. A similar trend was observed in prior studies on cells infected with vesicular stomatitis virus and bovine ephemeral fever virus, revealing a conserved pattern of PBLD suppression during viral infection.^[^ ^15^ ^]^ Functional studies have demonstrated that PBLD deficiency significantly enhanced viral infectivity, as evidenced by the increased susceptibility of *Pbld*‐KO mice to HAdV‐4 infection compared to their WT littermates. Mechanistically, we found that viral infection modulated TFEB expression in a growth phase and time‐dependent manner. TFEB, a member of the MITF family along with TFE3, is recognized as a vital transcriptional regulator of autophagy‐ and lysosome‐related gene expression. Our results indicate that TEFB acts as a regulator of *PBLD* transcription during viral infection, which is consistent with a previous report identifying TEFB as the master regulator of PBLD expression upon rapamycin induction.^[^ ^24^ ^]^ Furthermore, we demonstrated that viral infection upregulated the E3 ligase MARCH2, which catalyzes K48‐linked ubiquitination and subsequent degradation of the PBLD protein. These data suggest that PBLD downregulation by viral infection represents an evolutionarily conserved strategy to subvert host antiviral defenses and enhance viral fitness. However, the specific molecular mechanisms underlying this regulation require further investigation.

Autophagy is a conserved intracellular degradation system that maintains cellular homeostasis through lysosomal processing of cytoplasmic components, including protein aggregates, damaged organelles, and invading pathogens.^[^ ^31^ ^]^ Accumulating evidence demonstrates that autophagy is highly selective in delivering specific substrates, including essential sensors and adaptors of the type I IFN signaling response, for degradation through defined cargo receptors, thereby playing a predominant role in maintaining immune homeostasis.^[^ ^18^, ^32^ ^]^ In this study, we found that PBLD suppressed STING autophagic degradation to enhance the antiviral type I IFN response and identified the critical roles of the autophagic cargo receptor CCDC50 in mediating STING recognition. Recent studies have also reported that other autophagy cargo receptors, such as p62/SQSTM1, TOLLIP (toll interacting protein), and the endosomal sorting complexes required for transport (ESCRT)‐driven microautophagy plays an essential roles in regulating STING degradation.^[^ ^33^, ^34^, ^35^, ^36^, ^37^ ^]^ These findings identify autophagic degradation as an important mechanism in the regulation of STING expression.

Cargo receptors are well known to primarily recognize ubiquitination modifications on substrates to promote degradation in the autolysosome.^[^ ^38^ ^]^ Both K48‐ and K63‐linked ubiquitination are closely associated with STING regulation. For example, the E3 ubiquitin ligases tripartite motif proteins TRIM56 and TRIM32 catalyze K63‐linked polyubiquitination of STING, whereas ring finger protein 5 and TRIM30α dampens STING proteasomal degradation.^[^ ^39^, ^40^, ^41^, ^42^ ^]^ In this study, we found that PBLD reduced the K48‐linked ubiquitination of STING recognized by CCDC50, thereby inhibiting its autophagic degradation. This observation differs from previous reports, indicating that CCDC50 delivers K63‐polyubiquitinated STING and RIG‐I/MDA5 for autophagic degradation during viral infection.^[^ ^20^, ^43^ ^]^ These results suggest that CCDC50 recognizes diverse ubiquitin modifications (K48‐, or K63‐linked, and site‐specific) on STING to drive its autophagy‐dependent degradation. However, up to date, the specific E3 ligases or deubiquitinases responsible for the distinct types of ubiquitination modifications on STING recognized by CCDC50 remain unclear and warrant further investigation. Furthermore, CCDC50 knockdown effectively blocked DNA virus‐induced STING degradation in PBLD‐KO cells; however, silencing PBLD using siRNA in CCDC50‐KO cells did not fully restore STING protein levels or IRF3 phosphorylation. This indicates that CCDC50‐mediated autophagic degradation is not the sole pathway through which PBLD regulates STING. Notably, we observed that polyubiquitination of STING at Lys150 was driven by UBA52 in the absence of PBLD, serving as a recognition signal for the cargo receptor CCDC50. *UBA52* is a hybrid gene encoding a fusion protein comprising ubiquitin at the N‐terminus and ribosomal protein L4 (RPL40) at the C‐terminus. Upon translation, UBA52 is cleaved into RPL40 and ubiquitin, both of which play a critical roles in ubiquitination.^[^ ^44^, ^45^, ^46^ ^]^ Moreover, UBA52 facilitates the ubiquitination of the chaperone HSP90 together with E2 and E3 ligases, forming a complex that plays a critical role in maintaining homeostatic protein turnover.^[^ ^47^ ^]^ The cleavage of RPL40 from UBA52 is known to play multiple extra‐ribosomal roles, including protein synthesis.^[^ ^48^ ^]^ Our results showed that UBA52 bound to STING, and the downregulation of STING induced by PBLD silencing was effectively rescued in UBA52‐KO cells. It is possible that PBLD KO‐induced UBA52 binding to STING may not only serve as a ubiquitin supplier to the ubiquitin pool but is also act as a regulator of STING ubiquitination, thereby accelerating its degradation. To the best of our knowledge, we demonstrate for the first time that UBA52 drives STING ubiquitination and promotes its autophagic degradation via the autophagy‐lysosomal pathway, thereby expanding our understanding of ubiquitin‐dependent protein clearance mechanisms.

Multiple studies using a TMPD‐induced SLE mouse model have demonstrated the critical role of cGAS–STING signaling in lupus pathogenesis.^[^ ^29^, ^49^ ^]^ Our findings showed that *Pbld*‐deficiency in Pristane‐induced lupus (PIL) mice resulted in diminished autoimmune phenotypes and responses, including attenuated pathological damage, downregulated STING expression, and decreased type I IFN production compared with those in WT mice. These observations indicate that PBLD‐upregulated STING signaling could potentially accelerate disease pathogenesis. Furthermore, we found that PBLD was significantly upregulated in the leukocytes of patients with SLE through an integrative analysis of several large‐scale gene expression profiles from four SLE cohorts and clinical blood samples from a subset of patients. In addition, a strong positive correlation was observed between PBLD and the type I IFN signature in SLE, highlighting that aberrant PBLD expression may be a key pathogenic driver in this disease. Intriguingly, increased mRNA levels of *STING* and *TFEB*, along with decreased mRNA levels of *MARCH2*, were also observed, suggesting that transcriptional regulation of TFEB and MARCH may contribute to PBLD expression and promote STING expression in SLE. Consistent with this, downregulation of CCDC50 expression, which enhances STING expression, has been implicated in the pathogenesis of SLE.^[^ ^20^ ^]^ STING has been shown to be responsible for inducing the TFEB activation.^[^ ^50^ ^]^ Moreover, TFEB itself can also induce PBLD expression.^[^ ^24^ ^]^ This suggests a potential positive feedback loop between TFEB and PBLD that may regulate the STING‐mediated immune response. Clinically, inhibition of type I IFNs production has been regarded as a promising strategy for the treatment of SLE.^[^ ^51^ ^]^ Collectively, these results suggest that PBLD may serve as a promising therapeutic target for modulating aberrant type I IFN activity in patients with SLE. However, the molecular mechanisms underlying the role of PBLD in SLE pathogenesis require further investigation.

In conclusion, we identified PBLD as a positive regulator of STING‐induced type I IFN activation and elucidated the molecular regulatory network involved in PBLD‐mediated antiviral immune responses and autoimmune diseases. Based on our results, we propose that, under physiological conditions, PBLD is expressed at low levels in tissues and cells to maintain immune homeostasis by preventing the aberrant activation of the type I IFN pathway. During viral infection, PBLD expression is downregulated to subvert host antiviral defenses and promote viral replication. In contrast, under certain pathological conditions, PBLD expression is upregulated, which likely contributes to the development of autoimmune diseases such as SLE.

## 4 Experimental Section

### Cells, Viruses, and Mice

HeLa (RRID: CVCL_0030) and Madin‐Darby bovine kidney (MDBK, RRID: CVCL_0421) cells, obtained from the American Type Culture Collection (ATCC), were maintained in the laboratory. Mouse embryonic fibroblasts (MEFs) were prepared from pregnant females for 13‐day‐old embryos. Peritoneal macrophages (PMs) were acquired from the peritoneal lavage, and bone marrow‐derived macrophages (BMDMs) were isolated from the femur and tibia of *Pbld* ^+/+^and *Pbld* ^−/−^ mice, as described previously.^[^ ^16^ ^]^ All cells were cultured at 37°C in a 5% CO_2_ humidified incubator in Dulbecco's modified Eagle's medium (DMEM) or RPMI‐1640 medium, supplemented with 10% fetal bovine serum (FBS), 100 U mL^−1^ penicillin, and 100 µg mL^−1^ streptomycin. BMDMs differentiation was treated with GM‐CSF. *Pbld* ^−/−^ mice on a C57BL/6 genetic background have been described previously.^[^ ^17^ ^]^ All mice were housed in the specific pathogen‐free animal facility of the Shandong Normal University Animal Research Center with 50% to 60% humidity and a 12 h light/dark daily cycle. All animal experiments were conducted in accordance with protocols approved by the Institutional Animal Care and Use Committee of Shandong Normal University (Approval No. AEECSDNU2023023). The HSV‐1 and human adenovirus type 4 (HAdV‐4) used in this study have been previously described.^[^ ^16^, ^52^ ^]^

### Antibodies and Reagents

The following primary antibodies were used for immunoprecipitation, western blotting and immunofluorescence: β‐actin (Abways, AB0035), STING (Abways, BY9021), SQSTM1 (Abways, CY5546), p‐IRF3 (Abways, CY6305), UB (Abways, CY5520), UB‐K48 (Abways, CY5964), UB‐K63 (Abways, CY6579), UBA52 (Abways, DY1616), IgG (Abways, CY6900), Myc (Abways, AB0001), DYKDDDDK Tag (CST,14793), HA Tag (CST, 3724), PBLD (Santa Cruz, sc‐101502; Proteintech, 68317‐1‐Ig), cGAS (Santa Cruz, sc‐515802), TFEB (Aladdin, Ab130770), MARCH2 (Boster, A13497), GFP (Abclonal, AE011), CCDC50 (ABclonal, A17836), ATG7 (Abways, CY5658). The following secondary antibodies were used for HRP‐conjugated western blotting or immunofluorescence: Goat anti‐rabbit IgG (H+L) (Jackson, 111‐005‐003), goat anti‐mouse IgG (H+L) (Jackson,115‐005‐003), goat anti‐mouse IgG (H+L) Alexa Fluor 594 (ThermoFisher, A‐11005). Antibody details and reagent information are provided in Table S1 (Supporting Information).

### siRNA and DNA Oligonucleotides

siRNAs targeting PBLD, TFEB, or MARCH2 were synthesized and transfected with Lipofectamine 2000 according to the manufacture's protocol. The sequences of the 45‐nucleotide‐long sense and antisense strands of interferon‐stimulatory DNA (ISD) ISD45, HSV60, DNA90, and HSV120 oligonucleotides used in this study have been described previously.^[^ ^53^ ^]^ All oligonucleotides were synthesized by Sangon Biotech (Shanghai, China), and the sequences are listed in Table S2 (Supporting Information).

### Plasmid Construction and Generation of Stable Cell Lines

Specific PCR products corresponding to STING and its ubiquitination‐site mutants, CCDC50, TFEB, MARCH2, UBA52, UBA52 truncations, and cleavage‐resistant UBA52(G75/76A) were amplified using the primer pairs and subsequently cloned into appropriate vectors, as shown in Table S2 (Supporting Information). Guide RNA (gRNA) sequences targeting PBLD, CCDC50, and UBA52, listed in Table S2 (Supporting Information), were designed using the online browser CRISPR Direct (http://crispr.dbcls.jp/) and cloned into the lenti‐CRISPR/Cas9‐V2 system. PBLD‐overexpress HeLa cells, MDBK cells, PBLD‐ and CCDC50‐ knockout (KO) cell lines were generated following previously described methods.^[^ ^15^ ^]^

### Real‐Time Quantitative Polymerase Chain Reaction (RT‐qPCR)

Total RNA was extracted from cells or tissues using RNA extraction kit (Vazyme, RC102‐01). Equal amounts of total RNA (1 µg) were reverse transcribed into cDNA with PrimeScript RT Master Mix (Accurate, AG11706). qPCR was performed using SYBR Green qPCR Master Mix (ABclonal, RK21203). Gene expression levels were normalized to β‐actin, and relative expression was calculated by 2^−ΔΔCt^ method. A list of the primers used for qPCR analysis were synthesized by Tsingke Biotech (Beijing, China), with their sequences listed in Table S3 (Supporting Information).

### ELISA Assay

The ELISA kits (Multisciences, Lianke, EK2236) were used to detect the IFN‐β production in serum samples according to the manufacturer's instructions.

### Co‐Immunoprecipitation (Co‐IP) and Immunoblot Analysis

Co‐IP was performed following previously established methods.^[^ ^17^ ^]^ In brief, cells were lysed in ice‐cold cell lysis buffer (Beyotime, P0013) supplemented with 1 × protease and phosphatase inhibitor cocktail. The supernatant was incubated with appropriate antibodies or control IgG overnight at 4 °C, followed by precipitated with protein G/A agarose (Smart‐Lifesciences, SM009001) at room temperature. Beads were resuspended in protein loading buffer, and proteins were separated by SDS‐PAGE. Protein bands were transferred to polyvinylidene fluoride (PVDF) membranes (LABSELECT, TM‐PVDF‐R‐45) and probed with primary and secondary antibodies. Protein bands were detected using enhanced chemiluminescence (ECL) reagents (Shandong Sparkjade Biotechnology, ED0015‐C) on an imaging system (Tanon Science & Technology Co., Ltd., Shanghai, China).

For general western blot analysis, cells were lysed in RIPA buffer supplemented with protease and phosphatase inhibitors. Protein concentrations were determined using a bicinchoninic acid (BCA) protein assay kit (Yeasen, 20201ES).

### Immunoprecipitation Coupled with LC‐MS/MS Analysis

Total proteins were extracted from cells and subjected to immunoprecipitation (IP) using STING antibody and Dynabeads Protein G/A agarose beads as previously described above. STING‐bound proteins from wild‐type and PBLD‐KO cells were resolved in the gel and stained with Coomassie Blue (Beyotime). Then, protein bands were sent to the BGI Co., Ltd. (Beijing, China) for LC‐MS/MS analysis. Mass spectrometry data were processed using MaxQuant software (V1.6.6), and the database search algorithm Andromeda was used to search the Human's Proteome Reference Database from UniProt (2020‐05‐10). Finally, candidate STING‐interacting proteins were screened based on peptide score thresholds.

### Immunofluorescence (IF) Analysis

Cells were seeded on coverslips in 24‐well plates and transfected with the indicated plasmids for 24 h. Cells were fixed with 4% paraformaldehyde for 10 min, followed by permeabilization for 15 min at room temperature. After blocking with 5% BSA, samples were incubated with primary antibodies against target proteins and subsequently with fluorescently labeled secondary antibodies. Following counterstaining the nuclei with DAPI (SparkJade, EE0011‐A) for 5 min, the cells were mounted and imaged using a Leica SP8 confocal laser scanning microscope (Leica Microsystems, Germany). ImageJ software was used for the processing and analysis of images.

### Molecular Docking

Amino acid sequences of human PBLD (P30039‐1) and human MARCH2 (Q9P0N8‐1) were retrieved from the UniProt database (https://www.uniprot.org) and submitted to the AlphaFold 3 platform (https://alphafoldserver.com) for interaction prediction. The resulting interaction data were downloaded, and the CIF files contained within the data folder were imported into PyMOL 2.5.5 for visualization and analysis. Within PyMOL, potential docking hydrogen bonds were identified, and the overall quality of the docking results was assessed based on established criteria.

### Virus Challenge, TCID_50_ Assay, and Drug Treatment

These experiments were performed as previously described.^[^ ^16^ ^]^ Briefly, cells were infected with HAdV‐4 or HSV‐1 at an MOI of 2 for the indicated time. Subsequently, cell samples were collected for RT‐qPCR, western blot, or TCID_50_ assay. To determine viral titers, the samples underwent three cycles of freeze‐thawing and were subsequently subjected to TCID_50_ assay in 96‐well plates and calculated by the Reed‐Muench method, expressed as lgTCID_50_/mL. For drug treatments, cells were transfected with indicated plasmids or siRNAs for 36 h. After that, DMSO, MG132 (10 µg mL^−1^, MCE, HY‐13259C), Z‐VAD‐FMK (20 µm, SparkJade, SJ‐BP0011), or CQ (50 nm, MCE, HY‐12320) were added to the cells for 4 h. The medium was replaced, and cells were infected with HSV‐1 in the presence of the corresponding drug and collected at indicated time points.

### Pristane (TMPD)‐Induced SLE Mice Model

Eight‐week‐old *Pbld*‐deficient and *Pbld* ^+/+^ mice were intraperitoneally injected with 0.5 ml pristane (Aladdin, P106824) per mouse.^[^ ^54^ ^]^ Control mice received 0.5 ml mineral oil (Beyotime, ST275). Four months post‐injection, mice' urine was collected for creatinine and urea nitrogen analysis using a creatinine assay kit (Njjcbio, C011‐2‐1) and urea nitrogen detection kit (Njjcbio, C013‐2‐1), respectively. Mice were sacrificed, and PMs, BMDMs, kidney, and spleen samples were collected.

### Integrative Analysis of Gene Expression Profiles of SLE Cohorts

To comprehensively examine PBLD expression in SLE patients, four previously published GEO datasets about large‐scale gene expression profiles of leukocytes and PBMCs of 902 SLE patients and 116 healthy donors (HD) were downloaded, including GSE112087 (62 SLE, 58 HD), GSE72509 (99 SLE, 18 HD), GSE49454 (157 SLE, 20 HD) and GSE121239 (584 SLE, 20 HD).^[^ ^55^, ^56^, ^57^, ^58^ ^]^ Raw count matrix or FPKM gene expression matrix was downloaded. For further analysis, gene expression values from both data sets were FPKM normalized and log2 transformed. Principal component analysis (PCA) was used to reduce the dimension of the gene expression matrix and exclude outlier samples. The R “ggplot2” package was used to generate images. Gene correlations were tested by Pearson correlation and visualized as a Beeswarm, genetic relevance, and gene set enrichment analysis (GSEA).^[^ ^59^ ^]^ All statistical analyses were performed using the R/Bioconductor (version 4.4.2).

### Human Blood Samples

Human blood samples (plasma) were acquired from 9 diagnosed SLE patients at the Second Hospital, Cheeloo College of Medicine, Shandong University. Blood samples from age‐ and gender‐matched healthy donors were used as controls. The study was approved by the Committee for Ethical Review of Research involving Human Subjects of the Second Hospital, Cheeloo College of Medicine, Shandong University (KYLL2024301). All patients were informed of the study and provided written informed consent.

### Statistical Analysis

Data were presented as mean±standard deviation (S.D.) from at least three independent experiments. Statistical analyses were performed using GraphPad Prism 8 software. Protein band intensities were quantified using ImageJ software (https://imagej.net). The number of independent biological experiments and replicates (n) were indicated in the figure legends. Student's *t*‐test, one‐way analysis of variance (ANOVA), or two‐way ANOVA, as appropriate, were used to determine significant differences between groups. All statistical tests, *P*‐value< 0.05 is considered significant. Survival data were analyzed by Kaplan‐Meier curves with log‐rank test.

## Conflict of Interest

The authors declare no conflict of interest.

## Author Contributions

P.H. and H.Z. are co‐first authors and contributed equally to this work. H.B.H. and H.M.W. developed the concept of the study; H.B.H. and P.L.H. designed the experiments; H.C.Z., X.N.S., Y.Y.F., X.Y.W., and F.Z.Z. performed the experiments; R.L. and X.N.S. participated in the viral infection experiments; J.W., C.H.W., and Y.Y.H. helped construct the recombinant plasmids. H.C.Z., X.N.S. and X.Y.W. performed the mouse experiments; N.Z., S.W. and X.X.Z. provided clinical blood samples; H.C.Z., X.Y.L. and X.Y.Y. collected and analyzed the data; P.L.H. wrote the manuscript. H.M.W. and H.B.H. supervised the study. All the authors read and approved the final manuscript.

## Supporting information



Supporting Information

Supplemental Data 1

## Data Availability

The data that support the findings of this study are available from the corresponding author upon reasonable request.
