# Supplementary material for: PBLD Orchestrates the STING‐Mediated Antiviral Immune Response and Autoimmune Diseases
Source: Adv Sci (Weinh). 2025 Nov 8;13(4):e14512. doi: 10.1002/advs.202514512 (PMC12822414; doi:10.1002/advs.202514512)
Supplement: Supplementary file 1 — Supporting Information [file ADVS-13-e14512-s002.docx]

Supplementary Information

PBLD orchestrates the STING-mediated antiviral immune response and autoimmune diseases

*Peili Hou**^,†^, Hongchao Zhu^,†^, Xiaonan Sun, Ni Zhang, Song Wang, Xuexing Zheng, Xiaoyun Wang, Yueyue Feng, Fuzhen Zhang, Xingyu Li, Rui Li, Xiaomeng Wang, Yuanyuan Han, Jun Wang, Chuanhong Wang, Xiaoyang Yao, Hongmei Wang*, Hongbin He**

† These authors contributed equally: Peili Hou, Hongchao Zhu.

*Lead Contact

Correspondence: hongmeiwang@sdnu.edu.cn (H.W.); hongbinhe@sdnu.edu.cn (H.H.)

Correspondence and requests for materials should be addressed to:

H.W. ([hongmeiwang@sdnu.edu.cn](mailto:hongmeiwang@sdnu.edu.cn)); H.H. ([hongbinhe@sdnu.edu.cn](mailto:hongbinhe@sdnu.edu.cn))

Supplementary Figure 1-5

Supplementary Table 1, Table 2, Table 3

Description of Additional Supplementary Information

Supplementary Figure 1. PBLD deficiency attenuates DNA virus or interferon-stimulated DNA (ISD)-induced antiviral type I IFN response. Related to Fig.1.

Supplementary Figure 2. PBLD knockout/knockdown promotes STING autophagic degradation. Related to Fig.3.

Supplementary Figure 3. PBLD does not directly interact with STING to inhibit K48-linked ubiquitination of STING. Related to Fig.4.

Supplementary Figure 4. Viral infection downregulates PBLD by downregulation of TFEB and upregulation of MARCH2. Related to Fig.6.

Supplementary Figure 5. TMPD treatment attenuates the ubiquitination of STING in mice. Related to Fig.7.

Supplementary Table 1. List of key resources.

Supplementary Table 2. The Oligo sequences used in this study.

Supplementary Table 3. List of primers used in this study

Supplementary Figure 1


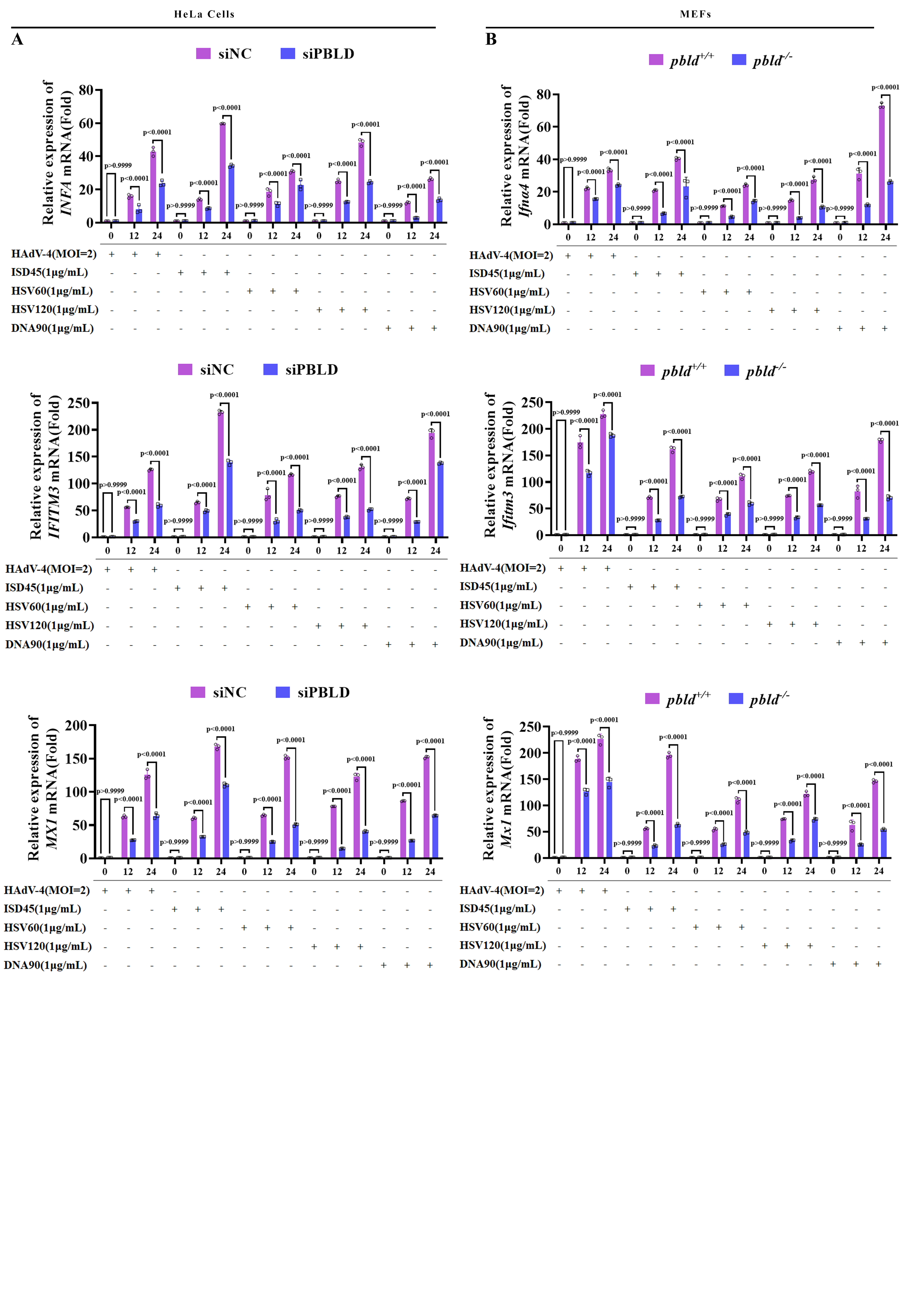


Fig.S1 PBLD Deficiency attenuates DNA virus or interferon-stimulated DNA (ISD)-induced antiviral type I IFN response

A) RT-qPCR assay detection of the mRNA levels of *IFNA* and ISGs (*IFITM3, MX1*) in control (siNC) and PBLD-silenced cells (siPBLD) in response to HAdV-4 infection or ISD45 (1 μg/mL), HSV60 (1 μg/mL), HSV120 (1 μg/mL) and DNA90 (1 μg/mL) transfection for the indicated time points. B) RT-qPCR analysis of *Ifna4,* *Ifitm3*, and *Mx1* mRNA expression in MEFs from *Pbld^+/+^* or *Pbld^-/-^* mice infected with HAdV-4 or stimulated with ISD45 (1 μg/mL), HSV60 (1 μg/mL), HSV120 (1 μg/mL) and DNA90 (1 μg/mL) for the indicated time points. Data in (A,B) are presented as mean±S.D., two-way ANOVA; n=3 biological independent experiments.

Supplementary Figure 2


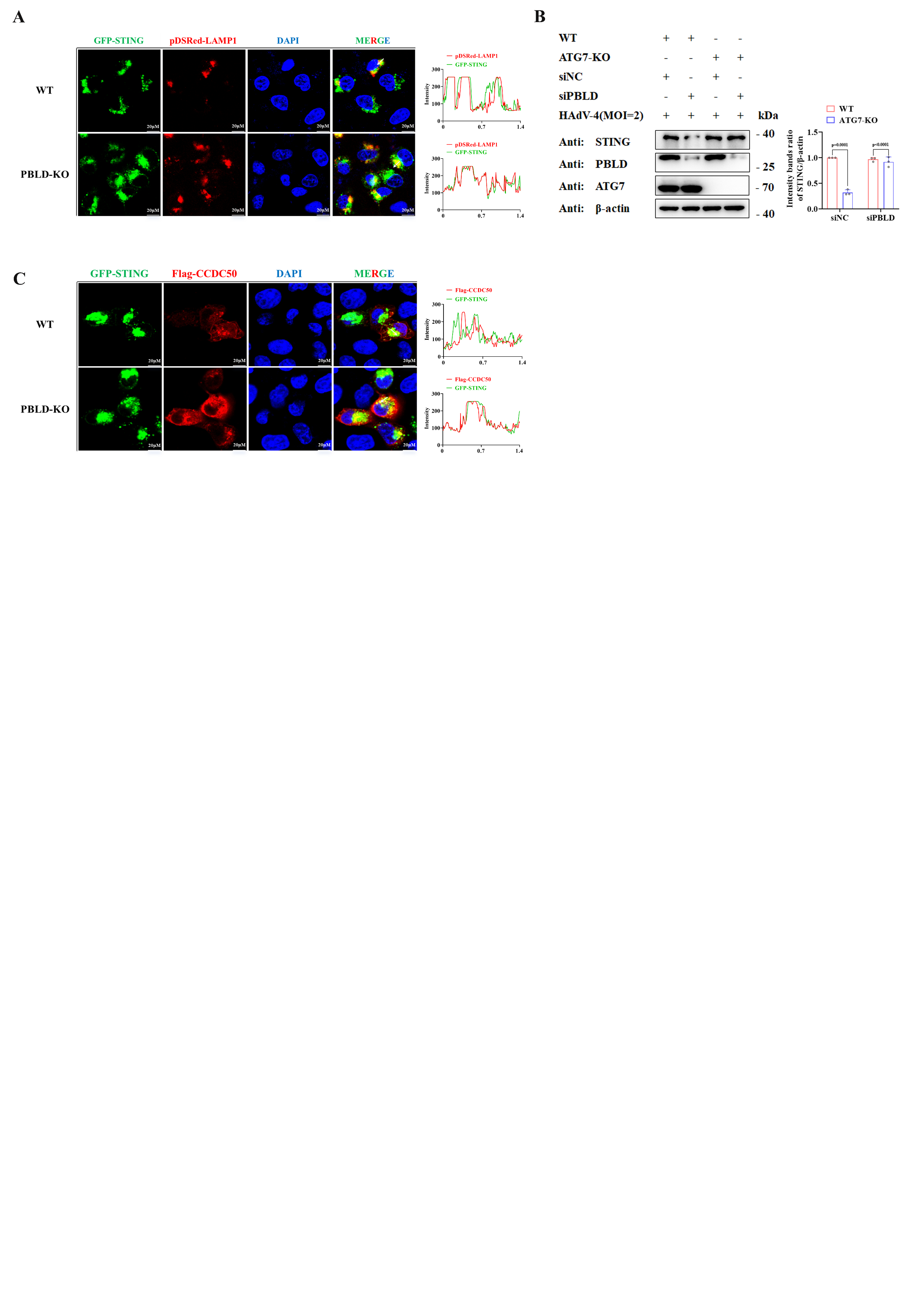


Fig.S2 PBLD knockout/knockdown promotes STING autophagic degradation

A) WT and PBLD knockout (PBLD-KO) HeLa cells transfected with GFP-STING and pDSRed-LAMP1 plasmids were incubated on a confocal dish, then treated with CQ (50 nM) for 12 h followed by HAdV-4 infection. Cells were stained with DAPI and observed by immunofluorescence microscopy to detect the colocalization of GFP-STING and pDsRed-LAMP1. Scale bar: 20 μm. Quantification of the colocalization between STING and LAMP1. B) WT and ATG7 knockout (ATG7-KO) HeLa cells were transfected with siNC or siRNAs for PBLD (siPBLD) in response to HAdV-4 infection, and the cell lysates were immunoblotted for the indicated protein. C) WT and PBLD knockout (PBLD-KO) HeLa cells transfected with GFP-STING and Flag-CCDC50 plasmids were incubated on a confocal dish, then infected with HAdV-4 in the presence of CQ (50 nM) for 12 h. Then the cells were stained with antibodies against Flag for CCDC50 and with DAPI for nucleus. Scale bar: 20 μm. Quantification of the colocalization between STING and CCDC50. Data in (A-C) are representative images. The intensity of the bands in data (B) was measured with the ImageJ software, and the results are quantifications from three independent experiments and presented as mean±S.D., two-way ANOVA.

Supplementary Figure 3


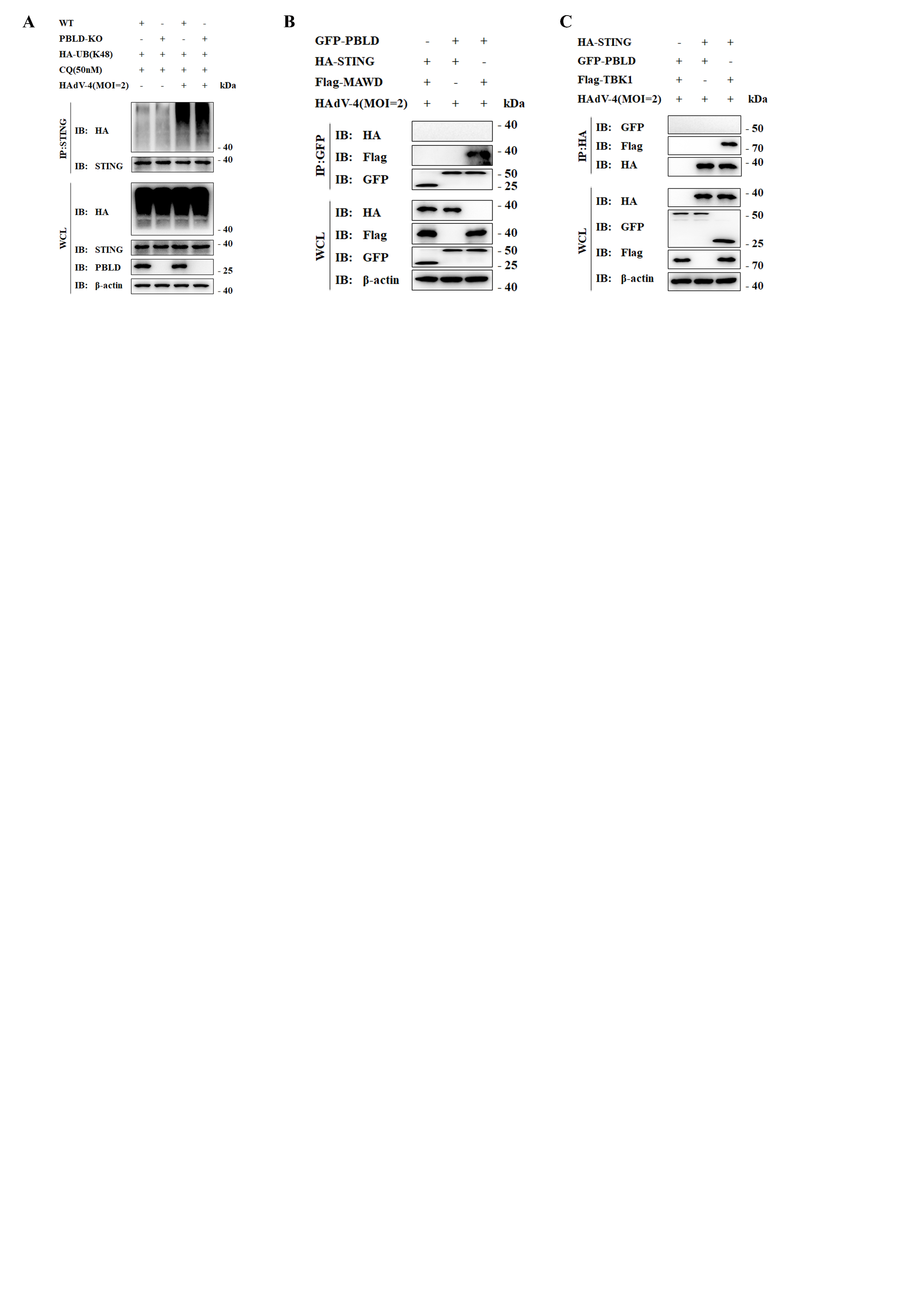


Fig.S3 PBLD does not interact with STING to inhibit K48-linked ubiquitination of STING

A) WT and PBLD knockout (PBLD-KO) HeLa cells transfected with HA-UB(K48) were treated with CQ (50 nM) followed by mock or HAdV-4 infection. Then the cells were lysed for immunoprecipitation with anti-STING and immunoblot analysis for STING ubiquitination. B, C) HeLa cells were transfected with the indicated plasmids followed by HAdV-4 infection. Then the cells were lysed for coimmunoprecipitation (co-IP) analysis, MAWD and TBK1 as positive controls for interaction with the PBLD and STING protein, respectively. Data in (A-C) are representative of three independent experiments.

Supplementary Figure 4


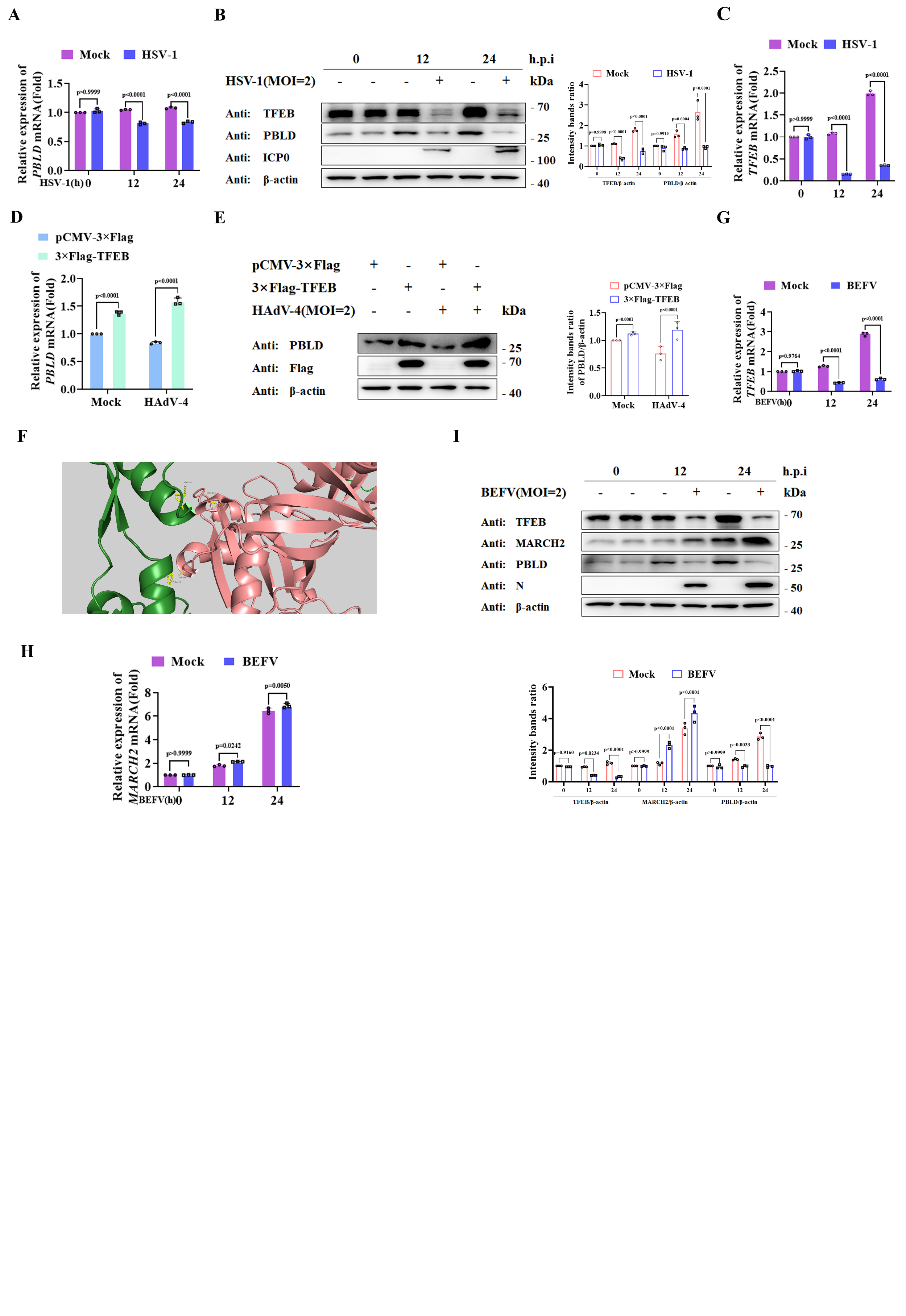


Fig.S4 Viral infection downregulates PBLD by downregulation of TFEB and upregulation of MARCH2

A, C) RT-PCR analysis of *PBLD*(A) and *TFEB*(C) expression in HSV-1-infected HeLa cells for the indicated time points. B) Western blot analysis of the indicated protein abundance after HSV-1 infection as the indicated MOI and time points. D, E) RT-qPCR analysis for the mRNA expression of *PBLD* (D) and immunoblot analysis for indicated protein expressions in HeLa cells (E). HeLa cells were transfected with TFEB-Flag or vector control and then treated with mock infection or HAdV-4(MOI=2) infection for 12 h. F) The interaction between March2 and PBLD was predicted by molecular docking. The PBLD is shown in pink color, MARCH2 protein is depicted in green, and the yellow dotted line represents the coordination interactions between PBLD and MARCH2 protein. G, H) Real-time PCR analysis of *TFEB* (G) and *MARCH2* (H) mRNA levels in BEFV-infected MDBK cells for the indicated time points. I) Immunoblot analysis the indicated protein expression in BEFV-infected MDBK cells for the indicated time points. Data in (A, C, D, G, H) are presented as mean±S.D., two-way ANOVA; n=3 biological independent experiments. Data in (B, E, I) are representative of three independent experiments. The intensity of the bands in data (B, E, I) were measured with the ImageJ software from three independent experiments and presented as mean±S.D., two-way ANOVA.

Supplementary Figure 5


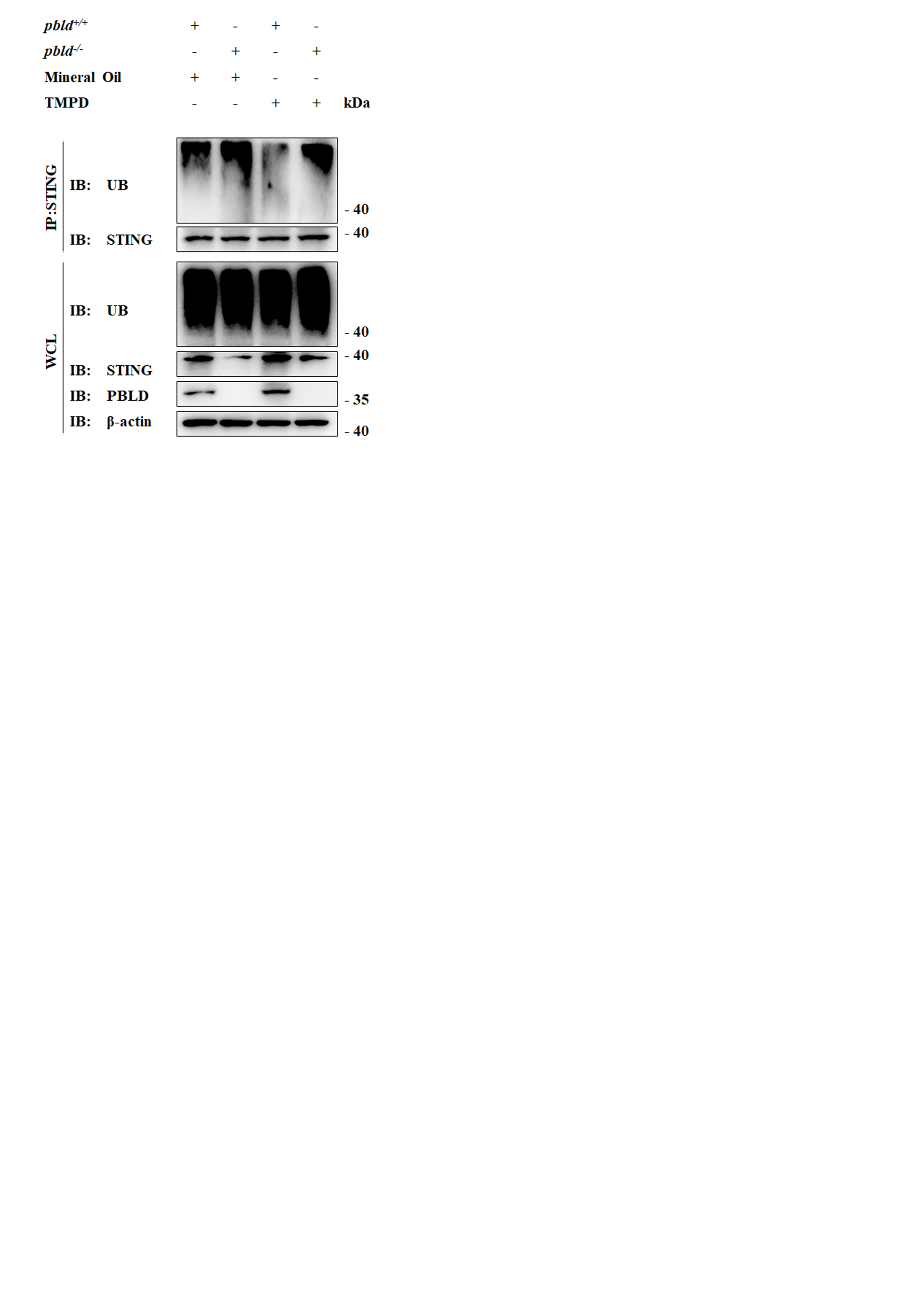


Fig.S5 TMPD treatment attenuates the ubiquitination of STING in mice

Ubiquitination analysis the STING protein of kidneys from 8-week-old mineral oil or TMPD-treated *Pbld*^+/+^ and *Pbld*^–/–^ mice. Data are representative of three independent experiments.

Supplementary Table 1. List of key resources

| Antibodies | Source | Identifier | Antibodies dilution |
| --- | --- | --- | --- |
| Mouse anti-HAdV-4-Penton base | Laboratory | N/A | 1:1000 |
| Rabbit anti-β-actin | Abways | AB0035 | 1:5000 |
| Rabbit anti-STING | Abways | BY9021 | 1:2000 (Human) |
| Rabbit anti-SQSTM1 | Abways | CY5546 | 1:2000 |
| Rabbit anti-p-IRF3 | Abways | CY6305 | 1:1000 |
| Rabbit anti-UB | Abways | CY5520 | 1:2000 |
| Rabbit anti-UB-K48 | Abways | CY5964 | 1:2000 |
| Rabbit anti-UB-K63 | Abways | CY6579 | 1:2000 |
| Rabbit anti-UBA52 | Abways | DY1616 | 1:2000 |
| Rabbit anti-IgG | Abways | CY6900 | 1:1000 |
| Rabbit anti-Myc | Abways | AB0001 | 1:2000 |
| Rabbit anti-ATG7 | Abways | CY5658 | 1:1000 |
| Rabbit anti-DYKDDDDK Tag | CST | 14793 | 1:1000 |
| Rabbit anti-HA Tag | CST | 3724 | 1:1000 |
| Mouse anti-PBLD | Santa Cruz | sc-101502 | 1:1000(Mouse) |
| Mouse anti-cGAS | Santa Cruz | sc-515802 | 1:2000 |
| Rabbit anti-TFEB | aladdin | Ab130770 | 1:2000 |
| Rabbit anti-MARCH2 | Boster | A13497 | 1:2000 |
| Rabbit anti-GFP | Abclonal | AE011 | 1:1000 |
| Rabbit anti-CCDC50 | ABclonal | A17836 | 1:2000 |
| Mouse anti-PBLD | Proteintech | 68317-1-Ig | 1:1000(Human) |
| Rabbit anti-TMEM173 | Affinity | DF12090 | 1:1000(Mouse) |
| Goat anti-rabbit IgG (H +L) | Jackson | 111-005-003 | 1:5000 |
| Goat anti-mouse IgG (H+L) | Jackson | 115-005-003 | 1:5000 |
| Goatanti-Mouse IgG (H+L), Alexa Fluor 594 | ThermoFisher | A-11005 | 1:2000 |
| Virus Strains | | |  |
| DH5α Competent *E. coli* | Tolobio | N/A |  |
| HAdV-4 | Henan Agricultural University | N/A |  |
| Herpes simplex virus type 1 (HSV-1) | Changchun Veterinary Research Institute | Kos strain |  |
| Bovine herpesvirus 1(BoHV-1) | Laboratory | N/A |  |
| Chemicals and Recombinant Proteins | | |  |
| MG132 | MedChemExpress | HY-13259C |  |
| Cycloheximide | MedChemExpress | HY-12320 |  |
| Z-VAD-FMK | SparkJade | SJ-BP0011 |  |
| Dimethyl sulfoxide (DMSO) | Sigma-Aldrich | 472301 |  |
| Chloroquine diphosphate salt (CQ) | Sigma-Aldrich | C6628 |  |
| Anti-Flag-tag Magnetic Beads | Biolinkedin | L-1011 |  |
| Anti-GFP-tag Magnetic Beads | Biolinkedin | L-1016 |  |
| Anti-HA-tag Magnetic Beads | MBL | M180-11 |  |
| Protein A/G magnetic Beads | ThermoFisher | 88803 |  |
| Attractene Transfection Reagent | QIAGEN | 301007 |  |
| Lipofectamine 3000 | Invitrogen | L3000015 |  |
| SYBR Green Fast qPCR Mix | ABclonal | RK21203 |  |
| T4 polynucleotide kinase | NEB | M0201S |  |
| Pristane | Aladdin | P106824 |  |
| Mineral oil | Beyotime | ST275 |  |
| Mouse IFN-β ELISA Kit | MULTI SCIENCES | EK2236 |  |
| Urea Assay Kit | Njjcbio | C013-2-1 |  |
| Creatinine (Cr) Assay kit | Njjcbio | C011-2-1 |  |
| DMEM | VivaCell | C3113-0500 |  |
| FBS | Umedium | 3021A |  |
| PBS | Sparkjade | CR0014 |  |
| Puromycin | Solarbio | P8230 |  |
| Mouse GM-CSF | Novoprotein | CK02 |  |
| Sparkjade ECL super | sparkjade | ED0015-C |  |
| ECL FemtoLight | epizyme | SQ202L |  |
| RIPA Buffer | New Cell and Molecular | WB3100 |  |
| NCM SDS-PAGE Loading Buffer | New Cell and Molecular | WB2001 |  |
| Halt^™^ Protease Inhibitor Cocktail, EDTA-Free (100×, P001) | Thermo Fisher Scientific | 78425 |  |
| Experimental Models: Cell lines |  |  |  |
| HeLa cells | ATCC (CCL-2) | CVCL_0030 |  |
| MDBK cells | ATCC (CCL-22) | CVCL_0421 |  |
| MEFs | In this study | N/A |  |
| BMDMs | In this study | N/A |  |
| PMs | In this study | N/A |  |
| Software and Algorithms |  |  |  |
| MEGA6 | Gene STAR | N/A |  |
| CRISPR direct | http://crispr.dbcls.jp/ | N/A |  |
| GraphPad Prism 7.0 | https://www.graphpad.com/scientific-software/prism/ | N/A |  |
| ImageJ | https://imagej.net/ij/ | N/A |  |
| GeneCards | https://previous.genecards.org | N/A |  |
| RStudio | https://www.r-project.org/ | N/A |  |
| PyMOL 2.5.5 | https://pymol.org/ | N/A |  |

Supplementary Table 2. The Oligo sequences used in this study

| Primer name | Sequence (5′-3′) | Application |
| --- | --- | --- |
| PBLD- Forward | CGGAATTCCG*gccacc*ATGGATTACAAGGATGACGACGATAAGAAGCTTCCTATTTTCATAG | pCMV-Flag-PBLD(Human) |
| PBLD- Reverse | GGGGTACCCCCTAGGCTGTCAGTGTGCCCTCT |  |
| Flag-STING-Forward | tggccatggaggcccgaattcGG*gccacc*ATGCCCCACTCCAGCCTGCAT | pCMV-Flag-STING(Human) |
| Flag-STING-Reverse | agatctcggtcgaccgaattcTCAAGAGAAATCCGTGCGGA |  |
| HA-STING-Forward | cgcgggcccaggcccgaattc*gccacc*ATGCCCCACTCCAGCCTG | pCMV-HA-STING(Human) |
| HA-STING-Reverse | agatctcggtcgaccgaattcTCAAGAGAAATCCGTGCGGA |  |
| Flag-STING(1-190aa)-Forward | tggccatggaggcccgaattcgg*gccacc*ATGCCCCACTCCAGCCTG | pCMV-Flag-STING (Human) -N |
| Flag-STING(1-190aa)-Reverse | agatctcggtcgaccgaattcTCATAGCAGGTTGTTGTAATGCTG |  |
| Flag-STING(K20R)-Forward | tggccatggaggcccgaattcGG*gccacc*ATGCCCCACTCCAGCCTGCATCCATCCATCCCGTGTCCCAGGGGTCACGGGGCCCAGAGGGCAGCCTTG | pCMV-Flag-STING(Human)- (K20R) |
| Flag-STING(K20R)-Reverse | agatctcggtcgaccgaattcTCAAGAGAAATCCGTGCGGA |  |
| Flag-STING(K137R)-Forward | AGCTGGGGCCAGGCCCCTGAGGCCCAGGAGGAT | pCMV-Flag-STING(Human)- (K137R) |
| Flag-STING(K137R)-Reverse | ATCCTCCTGGGCCTCAGGGGCCTGGCCCCAGCT |  |
| Flag-STING(K150R)-Forward | GCAGTGTGTGACGACGGGAATTTCAACGTG | pCMV-Flag-STING(Human)- (K150R) |
| Flag-STING(K150R)-Reverse | CACGTTGAAATTCCCGTCGTCACACACTGC |  |
| HA-CCDC50-Forward | cgcgggcccaggcccgaattc*gccacc*ATGGCTGAAGTCAGCATCGAC | pCMV-HA-CCDC50(Human) |
| HA-CCDC50-Reverse | agatctcggtcgaccgaattcGATGTTTGTAATGAAAACCTTTATGAGA |  |
| Flag-CCDC50-Forward | tggccatggaggcccgaattc*gccacc*ccATGGCTGAAGTCAGCATCGAC | pCMV-Flag-CCDC50(Human) |
| Flag-CCDC50-Reverse | agatctcggtcgaccgaattcTTAATGTTTGTAATGAAAACCTTTATGAG |  |
| HA-CCDC50(1-128aa)-Forward | cgcgggcccaggcccgaattc*gccacc*ATGGCTGAAGTCAGCATCGAC | pCMV-HA-CCDC50(Human)-N |
| HA-CCDC50(1-128aa)-Reverse | agatctcggtcgaccgaattcGCTTTCTCTTTTTCTCTTCCTGTAACT |  |
| GFP-UBA52-Forward | tcagatctcgagctcaagctt*gccacc*ATGCAGATCTTTGTGAAGACCCTC | pEGFP-UBA52(Human) |
| GFP-UBA52-Reverse | cgactgcagaattcgaagcttTTTGACCTTCTTCTTGGGACGC |  |
| GFP-UBA52(G75/76A)-Forward | TTGCGCCTGCGAGCTGCCATTATTGAGCCT | pEGFP-UBA52- (Human)- (G75/76A) |
| GFP-UBA52(G75/76A)-Reverse | AGGCTCAATAATGGCAGCTCGCAGGCGCAA |  |
| GFP-UBA52(1-76aa)-Forward | tcagatctcgagctcaagctt*gccacc*ATGCAGATCTTTGTGAAGACCCTC | GFP-UBA52(Human)-N |
| GFP-UBA52(1-76aa)-Reverse | cgactgcagaattcgaagcttGCCACCTCGCAGGCGCAA |  |
| GFP-UBA52(77-128aa)-Forward | tcagatctcgagctcaagctt*gccacc*ATGATTATTGAGCCTTCTCTCCGC | GFP-UBA52(Human)-C |
| GFP-UBA52(77-128aa)-Reverse | cgactgcagaattcgaagcttTTTGACCTTCTTCTTGGGACGC |  |
| 3×Flag-TFEB-Forward | aaggatgacgatgacaagctt*gccacc*ATGACAGCAAGCTCAGGCTGG | pCMV-3×Flag-TFEB(Human) |
| 3×Flag-TFEB-Reverse | tgaattcgcggccgcaagcttTCACAGCACATCGCCCTCC |  |
| Flag-MARCH2-Forward | tggccatggaggcccgaattc*gccacc*cATGACGACGGGTGACTGCTG | pCMV-Flag-MARCH2(Human) |
| Flag-MARCH2-Reverse | agatctcggtcgaccgaattcTCATACTGGTGTCTCCTCTGCCA |  |
| PBLD-gRNA(Human) | GCTGCTGTTTGCCTCCTAGA | Knockout of PBLD gene (Human) |
| PBLD-gRNA(Human) | TTGCCTCCTAGAAAATGAAT |  |
| PBLD-gRNA(Human) | CCTATTTTCATAGCAGATGCATT |  |
| PBLD-gRNA(Bovine) | GACTCATTCTCACCCTCAAGGGG | Knockout of PBLD gene (Bovine) |
| PBLD-gRNA(Bovine) | ACAATTTCCCTGCGTTCTGATGG |  |
| PBLD-gRNA(Bovine) | ATACTGTGCTCAGTAGCTACTGG |  |
| CCDC50-gRNA(Human) | GAACGTTCAGCGGAACCGTT | Knockout of CCDC50 gene (Human) |
| CCDC50-gRNA(Human) | TGTCCTGGAGGACCACACCC |  |
| CCDC50-gRNA(Human) | GTCCAAGCTGCCTGGAGTCA |  |
| UBA52-gRNA(Human) | TGGCAAAACCATCACCCTTG | Knockout of UBA52 gene (Human) |
| ATG7-gRNA(Human) | AGAAGTACCACTTCTACTAT | Knockout of ATG7 gene (Human) |
| ATG7-gRNA(Human) | TTTTAGTAGTGCCTTGGATG |  |
| ATG7-gRNA(Human) | GTGGGTTTGGATCAAAGGTT |  |
| siNC | UUCUCCGAACGUCACGUTT | Scrambled siRNA as negative control |
| siPBLD | GACUUGAUAAAGACUGCCAUA | Knockdown of PBLD gene (Human) |
| siTFEB | GCACAUUGGCUCCAACCCUTT | Knockdown of TFEB gene (Human) |
| siMARCH2 | GUGCUACCUUGCUUGAGAATT | Knockdown of MARCH2 gene (Human) |

Note: Lowercase English letters are homologous arm sequences; lowercase italicized letters are Kozak sequences; uppercase bold italics letters are mutated amino acid bases; uppercase underlined letters are Flag-tag sequences, and uppercase bold letters are protective bases and cleavage sites.

Supplementary Table 3. List of primers used in this study

| Primer name | Forward primer (5’ to 3’) | Reverse primer (5’ to 3’) |
| --- | --- | --- |
| β-actin(Human) | CGAGAAGATGACCCAGAT | GATAGCACAGCCTGGATA |
| IFNA(Human) | TCCCCGAGGAGGAGTTTGAT | TTCCACCCCAACCTCCTGTA |
| IFNB(Human) | CAGCAATTTTCAGTGTCAGAAGCT | TCATCCTGTCCTTGAGGCAGTAT |
| ISG15(Human) | AGCAGCTCCATGTCGGTGTCA | TGCGTCAGCCGTACCTCGTA |
| IFITM3(Human) | AATCACACTGTCCAAACCT | CTCCTCCTTGAGCATCTC |
| MX1(Human) | GTTTCCGAAGTGGACATCGCA | GAAGGGCAACTCCTGACAGT |
| TMEM173(Human) | CCTGAGTCTCAGAACAACTGCC | GGTCTTCAAGCTGCCCACAGTA |
| PBLD(Human) | TTGGGAGTTGAGCACCTTCG | TTACGTCTTCTGGGCTGACG |
| TFEB(Human) | CCTGGAGATGACCAACAAGCAG | TAGGCAGCTCCTGCTTCACCAC |
| MARCH2(Human) | CTAACACCAGCTACTGCGAGCT | GGAAACACACCATGTCGCAGCA |
| β-actin(Mouse) | CCACACCC*GCCACC*AGTTCG | TACAGCCCGGGGAGCATCGT |
| IFNA(Mouse) | ACCCACAGCCCAGAGAGTGACC | AGGCCCTCTTGTTCCCGAGGT |
| IFNB(Mouse) | CAGCTCCAAGAAAGGACGAAC | GGCAGTGTAACTCTTCTGCAT |
| ISG15(Mouse) | AGCCTCTGAGCATCCTGGTGAG | AGCGTGTCTACAGTCTGCGTCA |
| IFITM3(Mouse) | TGATCAACATGCCCAGAGATG | AGCCCAGGCAGCAGAAGTT |
| MX1(Mouse) | GAGTTCTTCTGGAGGATAGGA | TCTCGTTACTGTCTTCTGGT |
| ISG20(Mouse) | ACGCATTGAAGGAGGACA | ACGCTTGTGTTGGTTGAC |
| β-actin(Bovine) | GATGAGATTGGCATGGCTTTA | AACCGACTGCTGTCACCTTC |
| IFNA(Bovine) | GCACGAAGAACTGGATGATCAA | CGAGCCCTCTGTGCTGAAG |
| IFNB(Bovine) | CCTGTGCCTGA TTTCA TCA TGA | GCAAGCTGTAGCTCCTGGAAAG |
| ISG15(Bovine) | GGAGGCCCATGGATGATG | CCGAAGACGTAGATTCATGAACAC |
| IFITM3(Bovine) | CCTGACGACCACGGTGATC | CAGGCAGCACCAGTTCATGA |
| MX1(Bovine) | ATGGTTCATTCTGACTTG | TCTCTTCATATTGGCTGTA |
| HAdV-4-Pb | ATGATTATGATGGTAGTCAGGAT | ATGGTCACCGAGAAGTTG |
| BoHV-1-gE | CGTGGTGGTGCCAGTTAG | TCATCGTCGCTGTCGTCAT |
| HSV120 | AGACGGTATATTTTTGCGTTATCACTGTCCCGGATTGGACACGGTCTTGTGGGATAGGCATGCCCAGAAGGCATATTGGGTTAACCCCTTTTTATTTGTGGCGGGTTTTTTGGAGGACTT |  |
| DNA90 | TACAGATCTACTAGTGATCTATGACTGATCTGTACATGATCTACATACAGATCTACTAGTGATCTATGACTGATCTGTACATGATCTACA |  |
| HSV60 | TAAGACACGATGCGATAAAATCTGTTTGTAAAATTTATTAAGGGTACAAATTGCCCTAGC |  |
| ISD45 | TACAGATCTACTAGTGATCTATGACTGATCTGTACATGATCTACA |  |
